# *Candida albicans* Shaving to Profile Human Serum Proteins on Hyphal Surface

**DOI:** 10.3389/fmicb.2015.01343

**Published:** 2015-12-08

**Authors:** Elvira Marín, Claudia M. Parra-Giraldo, Carolina Hernández-Haro, María L. Hernáez, César Nombela, Lucía Monteoliva, Concha Gil

**Affiliations:** ^1^Departamento de Microbiología II, Facultad de Farmacia, Universidad Complutense de MadridMadrid, Spain; ^2^Unidad de Proteómica, Facultad de Farmacia, Universidad Complutense de MadridMadrid, Spain; ^3^Instituto Ramón y Cajal de Investigación SanitariaMadrid, Spain

**Keywords:** *Candida albicans*, shaving, surface proteins, human serum, complement pathways, coagulation pathways, GPI-anchored proteins, host-pathogen interaction

## Abstract

*Candida albicans* is a human opportunistic fungus and it is responsible for a wide variety of infections, either superficial or systemic. *C. albicans* is a polymorphic fungus and its ability to switch between yeast and hyphae is essential for its virulence. Once *C. albicans* obtains access to the human body, the host serum constitutes a complex environment of interaction with *C. albicans* cell surface in bloodstream. To draw a comprehensive picture of this relevant step in host-pathogen interaction during invasive candidiasis, we have optimized a gel-free shaving proteomic strategy to identify both, human serum proteins coating *C. albicans* cells and fungi surface proteins simultaneously. This approach was carried out with normal serum (NS) and heat inactivated serum (HIS). We identified 214 human and 372 *C. albicans* unique proteins. Proteins identified in *C. albicans* included 147 which were described as located at the cell surface and 52 that were described as immunogenic. Interestingly, among these *C. albicans* proteins, we identified 23 GPI-anchored proteins, Gpd2 and Pra1, which are involved in complement system evasion and 7 other proteins that are able to attach plasminogen to *C. albicans* surface (Adh1, Eno1, Fba1, Pgk1, Tdh3, Tef1, and Tsa1). Furthermore, 12 proteins identified at the *C. albicans* hyphae surface induced with 10% human serum were not detected in other hypha-induced conditions. The most abundant human proteins identified are involved in complement and coagulation pathways. Remarkably, with this strategy, all main proteins belonging to complement cascades were identified on the *C. albicans* surface. Moreover, we identified immunoglobulins, cytoskeletal proteins, metabolic proteins such as apolipoproteins and others. Additionally, we identified more inhibitors of complement and coagulation pathways, some of them serpin proteins (serine protease inhibitors), in HIS vs. NS. On the other hand, we detected a higher amount of C3 at the *C. albicans* surface in NS than in HIS, as validated by immunofluorescence.

## Introduction

The yeast *Candida albicans* is the most important opportunistic fungi, causing a wide variety of infections ranging from superficial to invasive candidiasis, and it is often found in the mucosal microbiota. *C. albicans* infections produce high morbidity and mortality in intensive care, post-surgery and cancer patients or other types of immunocompromised patients (Leleu et al., 2002; Almirante et al., 2005; Hube, 2006; Brown et al., 2012). The high mortality of these infections is determined by the diagnostic limitations, the scarcity of antifungal agents and the emergence of resistance to them (Viudes et al., 2002; Wilson et al., 2002). For these reasons, the incidence of invasive candidiasis is still high.

The cell wall of *C. albicans* is a dynamic and complex multi-layered structure located external to the plasma membrane. It is responsible for maintenance of the shape that characterizes each growth form (mainly yeast and hyphae) of the fungus (Klis et al., 2009). The cell wall mediates the first contact with the environment and integrates multiple cues into complex signaling networks that are coordinated by various transcription factors. Consequently, *C. albicans* differentially expresses cell surface proteins and virulence factors. The dimorphic transition that is the ability of *C. albicans* to reversibly switch from yeast to hyphal growth is essential for virulence; strains that are locked in either form are avirulent (Yan et al., 2013; Lu et al., 2014). The hyphal form allows the pathogen to penetrate into tissues to acquire nutrients or escape from the host defense, and yeast cells disseminate across the human body. There are many signals capable of inducing the dimorphic phenotypic switch: N-acetyl-D-glucosamine, physiological temperature and pH (37°C and neutral), high CO_2_ concentration, hypoxia and nutrient starvation; nonetheless, growing in human serum is the most physiological condition to study this process (Kumamoto and Vinces, 2005; Karkowska-Kuleta et al., 2009; Sudbery, 2011; Mayer et al., 2013; Ene et al., 2014).

*C. albicans* is able to adhere to host cells and tissues; for this reason, it exposes surface proteins such as adhesins and many other pathogenic factors (Chaffin, 2008). Adhesion to host tissue is a prerequisite for tissue invasion and infection. Among adhesins, there are the Als family (agglutinin-like sequence), the Hwp family and the Iff/Hyr family (de Groot et al., 2013). Pathogenic factors include tissue-digesting hydrolytic enzymes such as the Sap family (Secreted Aspartyl Proteinases) (Kretschmar et al., 2002; Buu and Chen, 2014), lipases and phosholipases. The Sap family is composed of 10 proteins with different expression patterns; they act optimally at acidic pH. Their main role is related to digesting molecules for nutrient acquisition and for digesting or distorting host cell membranes to avoid or resist antimicrobial attack by the host immune system (Naglik et al., 2003). Some adhesins are attached to the wall through a C-terminus glycosylphosphatidylinositol (GPI) anchorage sequence (De Groot et al., 2003; Pardo et al., 2004; Klis et al., 2009).

Human serum is a very complex body fluid; its qualitative and quantitative composition was a hot topic of study during years (Anderson and Anderson, 2002; Mitchell, 2010). In the Plasma Proteome Database, proteomic data of 10,546 proteins detected in human serum and plasma are collected (Nanjappa et al., 2014). The dynamic range of abundance of these proteins has more than 10 orders of magnitude of variation. The complement system is an efficient component of the innate immune response and establishes a link with the adaptive system. The three conventional pathways by which complement is activated (classical, alternative and lectin) are composed of around 30 proteins (Engleberg et al., 2012). It mediates several physiological processes such as tissue regeneration and clearance of immune complexes. C2 and factor B (FB) are the main heat-sensitive complement components (Soltis et al., 1979). C2 is the central component in the activation of classical and lectin pathways and FB is for alternative pathway; both are necessary for the formation of C3- and C5-convertases in the respective complement cascades aforementioned.

*C. albicans* develops several strategies to evade the complement system, delay the detection by the host immune systems and even capable of manipulate the host defenses for its own purpose (Collette and Lorenz, 2011). In this way, *C. albicans* acquires host-derived inhibitory proteins such as factor H (FH), factor H-like protein 1 (FHL-1) and C4b-binding protein (C4BP), to prevent activation of complement pathways on its surface. These three protein regulators are closely related molecules composed of variable numbers of short consensus repeat (SCR) domains. There are four *C. albicans* proteins described with the ability to fix FH on their surface: glycerol 3-phosphate-dehydrogenase 2 (Gpd2), high-affinity gluthathione transporter 1 (Hgt1), phosphoglycerate mutase 1 or complement regulator-acquiring surface protein 1 (Gpm1/CRASP1) and pH-regulated antigen 1 (Pra1/CRASP2) (Poltermann et al., 2007; Luo et al., 2009, 2013). With the exception of Hgt1, the other three are able also to bind plasminogen. Additionally, Pra1 and Hgt1 bind C4BP (Lesiak-Markowicz et al., 2011; Luo et al., 2011). Factor H mediates binding to polyanions (sialic acids and human heparin) present on *C. albicans* cell wall (Soares et al., 2000; Rodríguez de Córdoba et al., 2004; Green et al., 2013). Pra1 also binds fibrinogen and C3 directly (Zipfel et al., 2011).

The aim of this study is to know the human serum protein coat on *C. albicans* surface as well as which *C. albicans* proteins could be candidates for diagnostic markers, potential vaccines or therapeutic targets, as they are detected when human serum is present. The interaction between human serum proteins and other microorganism surface was tested by different proteomic approaches in *Staphylococcus aureus* and *Streptococcus pyogenes* (Dreisbach et al., 2011; Sjöholm et al., 2014). In this work, we carried out a gel-free proteomic approach by shaving live *C. albicans* cells with trypsin after serum interaction to obtain exposed peptides of *C. albicans* and human proteins. With this shaving strategy that was applied in our laboratory to *C. albicans* and *S. cerevisiae* cultured in synthetic media (Hernáez et al., 2010; Insenser et al., 2010; Gil-Bona et al., 2015), it is not necessary to perform sub-cellular fractionation. In the present work, we have optimized this protocol for the detection of *C. albicans* and human proteins together in conditions that mimic *in vivo* interaction during systemic infections; overall, 372 *C. albicans* and 214 human proteins were identified. Among *C. albicans* proteins, 147 are described as located at the cell surface. Interestingly Pra1 and Gpd2 are able to attach inhibitors of complement cascades and plasminogen; while a further 7 proteins are also capable of attaching plasminogen. Regarding human proteins, all main proteins of complement pathways were identified, and many proteins involved in coagulation and metabolism, as well as immunoglobulins or cytoskeletal proteins. Furthermore, in this work proteins of complement and coagulation pathways were observed surrounded the surface of *C. albicans* hyphal cells.

## Materials and methods

### *Candida albicans* strain and growth conditions

The *C. albicans* strain used in this work was SC5314, from a clinical isolate (Gillum et al., 1984). This strain was grown on YPD plates (2% D-glucose, 2% peptone, 1% yeast extract, and 2% agar) and incubated at 30°C. A colony was picked and grown in YPD medium at 30°C and 200 rpm during 6–8 h. The OD_600nm_ was measured and diluted on minimum medium (MM) at 0.00003 OD_600nm_ (2% D-glucose, 0.17% yeast nitrogen base, 0.5% (NH_4_)_2_SO_4_, 0.192% synthetic complete mixture (Kaiser) drop-out -ura and 0.01% uracil). Culture was grown at 30°C and 200 rpm overnight to obtain and collected at a concentration ~10^6^ cells/ml. 1.5 × 10^7^ cells were resuspended in 9 ml of Lee medium at pH 6.7 (Lee et al., 1975). One milliliter of human serum was added and the culture was grown during 5 h at 37°C and 200 rpm. Human sera were from healthy donor volunteers who did not have any clinical or microbiological evidence of infection. They were used in the experiments as normal serum (NS) or heat inactivated serum (HIS). Serum inactivation was done by heating at 56°C during 30 min in a water bath.

### Surface shaving

Surface shaving procedure was adapted from previous experiment of our group (Hernáez et al., 2010; Gil-Bona et al., 2015). Briefly, after incubation of *C. albicans* with 10% human serum (normal serum-NS or heat inactivated serum-HIS), the cultures were centrifuged at 3500 rpm 5 min. Cell pellet was resuspended in 1 ml of phosphate buffer saline (PBS) with 0.1% Tween 20, centrifuged again and washed 6 times more with PBS. The last pellet was resuspended in 400 μl of ammonium bicarbonate (AMBI buffer; NH_4_HCO_3_) 25 mM pH 8.0 and 7.5 μl dithiothreitol (DTT) 1 M and 9 μg of recombinant sequencing grade trypsin (Roche) were added. DTT was added during the digestion as reducing agent to consent deep digestion of non-covalently or disulphide bridges associated protein. The samples were incubated at 37°C, 30 min and 600 rpm. After incubation, samples were centrifuged at 5000 rpm 5 min and the supernatant was filtered with a filter unit of 0.22 μm. The cell pellet was resuspended in 400 μl of fresh AMBI buffer and 100 μl of trifluoroacetic acid (TFA) 0.1% (v/v) were added to stop the proteolytic reaction. It was centrifuged and the supernatant was filtered again. Both peptide-supernatants were put together and processed for further proteomic analysis. Subsequently, originated peptides were cleaned up with a Poros R2 resin (ABSciex, Framingham, MA). Peptides were eluted with 80% acetonitrile in 0.1% TFA, dried in a Speed-Vac and resuspended in 0.1% formic acid. The samples were stored at −20°C before nano-LC-MS/MS analysis.

### LTQ-Orbitrap velos analysis, protein identification, and bioinformatics analysis

Peptides were analyzed by nano-LC-MS/MS analysis. Peptide samples were concentrated and desalted using C18-A1 ASY-column 2 cm pre-column (Thermo Scientific) and then eluted onto a Biosphere C18 column (Nano-Separations). Peptides were separated with a 140 min gradient (110 min from 0 to 40% Buffer B; Buffer A: 0.1% formic acid/2% acetonitrile; Buffer B: 0.1% formic acid in acetonitrile) at a flow-rate of 250 nl/min on a nano-Easy HPLC (Proxeon) coupled to a nano-electrospray ion source (Proxeon). Mass spectrometry experiments were performed using a LTQ-Orbitrap Velos (Thermo Scientific) in the positive ion mode. Full-scan MS spectra (m/z 400/1400) were acquired in the Orbitrap apparatus with a target value of 1,000,000 at a resolution of 60,000 at m/z 400 and the 15 most intense ions were selected for collision induced dissociation (CID) fragmentation in the LTQ with a target value of 10,000 and normalized collision energy of 38%. Precursor ion charge state screening and monoisotopic precursor selection were enabled. Singly charged ions and unassigned charge states were rejected. Dynamic exclusion was enabled with a repeat count of 1 and exclusion duration of 30 s.

Protein identification was carried out with mass spectra raw files using a licensed version of search engine MASCOT 2.3.0 with Proteome Discoverer software version 1.4.1.14 (Thermo Scientific). Searchers were made against *C. albicans* SC5314 (6221 sequences) and human database (20233 sequences) available on *Candida* Genome Database (Assembly 21 of CGD, http://www.candidagenome.org/) and Uniprot-SwissProt (http://www.uniprot.org) respectively, to identify peptides and proteins. Search parameters were oxidized methionine as variable modification, peptide mass tolerance 10 ppm, 2 missed trypsin cleavage sites and MS/MS fragment mass tolerance of 0.8 Da. In all protein identification, the FDR was <1%, using a Mascot Percolator, with a *q*-value of 0.01. Gene Ontology (GO) terms at CGD (http://www.candidagenome.org/cgi-bin/GO/goTermFinder) were used to classify *C. albicans* proteins.

As an estimation of the relative protein abundances the normalized spectral abundance factor (NSAF) was used (Zybailov et al., 2007), and the average of the normalized values was calculated. Mass spectrometry proteomics data have been deposited in *C. albicans* PeptideAtlas (Vialas et al., 2014) with the data set identifier PASS00446.

### Cell permeability

Before and after trypsin treatment, *C. albicans* cell wall permeability was evaluated by staining with propidium iodide (PI). 10^6^ cells/ml were incubated with 10 μl of PI 5 mM and the positive fluorescent cells (red staining) were checked, at least 200 cells of each sample were counted in a fluorescence microscope. Cells treated with 70% ethanol-PBS (v/v) were used as positive control.

### Epifluorescence and confocal fluorescence microscopy

For detecting complement proteins on *C. albicans* surface after interaction with human serum, a solution of 10^6^ cells/ml on Lee medium at pH 6.7 with 10% of human serum (NS or HIS) was prepared. 10^5^ cells of this suspension were laid during 30 min, 1.5 or 5 h at 37°C on glass coverslips pre-coated with poly-L-lysine (1 mg/ml). Cells were fixed with formaldehyde 4% (w/v) (in PBS) for 20 min at room temperature (RT). Glass slides were washed twice with PBS. To stain cell membrane, PKH26 dye was added following technical instructions (Sigma Aldrich). Then, slides were blocked during 45 min at RT with buffer B (PBS plus bovine serum albumin (BSA) at 1 mg/ml). The slides were washed twice again with PBS and then incubated for 1.5 h at RT in the same buffer with an anti-C3, anti-factor B polyclonal serum (dilution 1:75 and 1:50, respectively) or only buffer. The slides were washed three times with PBS and further incubation for 1 h with an anti-rabbit IgG conjugated with Alexa-488 diluted at 1:500 in buffer B was done. Nuclei were stained with DAPI dye (5 μg/ml; 5 min at RT). Mounting medium Fluoromount-G (SouthernBiotech) was added to the preparations. Cells were then examined with epifluorescence and confocal microscopy images were collected using an Olympus FV1200 microscope.

### Flow cytometry analysis

*C. albicans* cells were grown as previously described on Immunofluorescence assay section. In this case the incubation with human serum (NS or HIS) was done during 30 min to avoid larger hyphae formation. After that, cells were washed with PBS and fixed with formaldehyde 4% in PBS during 20 min at RT. Then, three washes with PBS were performed and samples were blocked with 0.1% BSA in PBS at 4°C overnight with gentle shaking. Samples were incubated with primary antibody or with buffer alone as negative control, for 2 h at RT. The primary antibodies were prepared in PBS with 0.1% BSA, anti-C3 and anti-FB at 1:75 and 1:50 dilutions, respectively. Four PBS washes were performed and incubated with secondary antibody below, an anti-rabbit conjugated with Alexa-488 diluted at 1:500, 2 h at RT with gently shaking. Then, samples were washed with PBS 4 times more. These cells were analyzed with a Guava easyCyte cytometer of Millipore.

## Results

### Gel-free proteomic approach to study protein interactions between *C. albicans* and human serum

In order to break through the broad spectrum of human serum proteins attached to *C. albicans* cells and the fungus surface proteins, we analyzed *C. albicans* cell surface after 5 h of incubation with 10% human serum by a gel-free proteomic strategy. Normal serum (NS) was used to mimic physiological conditions of *C. albicans* during invasive candidiasis and heat inactivated serum (HIS) to determine human proteins deposited on the *C. albicans* surface without conventional complement cascade activation. As human serum is the most important inductor of *C. albicans* dimorphic transition, after 5 h of incubation all *C. albicans* cells were in the hyphal form. To obtain the protein peptides, a shaving methodology was used, which consists of direct cell digestion with trypsin and MS/MS identification. Before digestion, cells were washed with PBS plus Tween-20 and several additional washes with PBS were done to remove human serum proteins weakly attached to *C. albicans* cells. We optimized parameters such as time and trypsin concentration during shaving of *C. albicans* cells, in order to control cell integrity and identify the highest number of proteins (from both, *C. albicans* and human). With short times of trypsin treatment, between 5 and 10 min, only human proteins were identified (data not shown). Otherwise, after 30 min, human and *C. albicans* proteins were detected; thus, this duration of trypsin incubation was selected. Before and after trypsin treatment, cells were treated with propidium iodide (PI) and the cell permeability state was analyzed under the microscope. Less than 1% positive staining was obtained in all conditions. Cell integrity was assessed with the purpose of avoiding cytoplasmic protein contamination after trypsin treatment.

Peptides obtained after *C. albicans* cells interaction with 10% human serum (NS or HIS) by shaving were recovered and identified by LC-MS/MS on a LTQ Orbitrap. Four biological replicates were made using NS and three using HIS. Mass spectra raw files were searched against *C. albicans* SC5314 and human databases (CGD and Uniprot-SwissProt, respectively) and proteins identified in at least two biological replicates with two or more peptides in one of them were selected. *C. albicans*-identified proteins are listed in Table 1 and Table S1, and human proteins in Table 2 and Table S2.

**Table 1:** **Selected ***C. albicans*** proteins identified after incubation with 10% NS or HIS related with cell surface and immunogenicity**.

**Categories**	**Common in NS and HIS**	**Only identified in NS**	**Only in HIS**	**Total**
GPI-anchored proteins^a^	11	12	–	23
	**Als3**, Cht2, Ecm33, **Hyr1**, Ihd1, Pga45, Phr1, Pir1, Rbt1, **Rbt5**, Sod5	**Als1**, Crh11, Pga10, **Pga4**, Phr2, Plb3, Plb4.5, Rhd3, Sap9, Ssr1, **Utr2**, Ywp1	–	
Proteins involved in cell wall organization or biogenesis^b^(GO: 71554)	8	18	–	26
	Act1, Bmh1, Gda1, Msb2, **Sod1,** Srb1, **Tsa1, Yps7**	Agm1, **Bgl2**, Eng1, Frs1, Gfa1, Gsc1, Kex2, Mnn26, Mnt1, Pmi1, Pmt2, Ras1, Rho1, Rvs161, Sim1, **Slk19,** Sur7, Ypt31	–	
Cell surface proteins^b^(GO: 9986)	40	21	1	62
	**Adh1, Cdc19,** Cdc48, **Cef3,** Ddr48, **Ece1**, Efb1, **Eft2,** Egd2, **Eno1**, **Fba1, Glx3, Hsp70, Hsp90, Ino1, Ipp1,** Kar2, **Met6,** Mp65, Orf19.2478.1, **Pdc11, Pdi1, Pgk1,** Pma1, Pra1, Rpl10, Rpl17B, Rpl3, Rpl4B, **Rps7A,** Rps8A, **Sam2, Ssa2, Ssb1, Tal1, Tdh3,** Tef1, **Tkl1, Tos1, Tpi1**	Ape2, **Atp1**, **Atp2**, Coi1, Csp37, Gpd2, Gph1, **Hem13**, Hsp104, Hsp21, Rpl13, Rpl14, Rpl19A, Rpl20B, Rpl6, Rps10, Rps6A, **Ssc1**, Ssz1, Ugp1, Xyl2	Cht3	
Plasma membrane proteins^b^ (GO: 5886)	8	28	–	36
	Ahp1, Orf19.6553, Cof1, Met15, Pfy1, **Pgi1,** Pil1, Tif	**Ade17,** Adh2, Bcy1, Bzz1, Cdc42, Chc1, Faa4, **Gca1**, Hgt6, Ist2, Kin2, Lsp1, Mdg1, Orf19.1564, Orf19.3003, Orf19.4216, Orf19.6160, Pet9, Pga63, Pst3, Rdi1, Sec26, Sec4, **Shm2,** Sso2, Tom70, Ypt1, Ykt6	–	
Others immunogenic proteins	6	2	–	8
	**Asc1, Gnd1, Grp2, Hxk2, Msi3, Sah1**	**Acs2, Mdh1**	–	
				155

The classification is hierarchical and exclusive. Proteins were included if they were detected in at least two replicates of one condition with at least 2 peptides in one replicate. There are four replicates of Normal Serum (NS) and three replicates of Heat Inactivated Serum (HIS). Protein names from Candida Genome Database (CGD) (Inglis et al., 2012).

a GPI-cell wall proteins reviewed in De Groot et al. (2003), Pardo et al. (2004), Chaffin (2008), Klis et al. (2009).

b Gene Ontology (GO) terms (in parentheses the GO identifier) at CGD.

Proteins able to induce human serum antibodies in patients with invasive candidiasis are indicated in bold (Pitarch et al., 2004, 2006, 2008, 2011; Mochon et al., 2010).

**Table 2:** **Human proteins of complement and coagulation pathways identified on the surface of ***C. albicans*** after incubation with human serum**.

**Accession number^a^**	**Gene symbol^a^**	**Description/Function^a^**	**No. replicates (peptides on each replicate)^b^**	
			**NS**	**HIS**
**COMPLEMENT PATHWAY**				
P01023	A2M	Alpha-2-macroglobulin	2 (15,11)	3 (10,9,70)
P04003	C4BPA	C4b-binding protein alpha chain	4 (5,3,4,9)	3 (15,8,4)
P20851	C4BPB	C4b-binding protein beta chain	1 (2)	2 (2,3)
P10909	Clusterin	Extracellular chaperone or ApoJ	4 (11,8,13,19)	3 (11,13,18)
Q9BWP8	COLEC11	Collectin kidney protein 1 or collectin-11	4 (5,5,2,4)	0
P02745	C1qA	Complement C1q subcomponent subunit A	0	2 (1,4)
P02746	C1qB	Complement C1q subcomponent subunit B	3 (1,1,1)	3 (5,5,4)
P02747	C1qC	Complement C1q subcomponent subunit C	3 (1,1,2)	3 (7,5,6)
P00736	C1r	Complement C1r subcomponent	2 (2,7)	3 (21,15,14)
Q9NZP8	C1r-like	Complement C1r-like protein	0	3 (6,5,5)
P09871	C1s	Complement C1s subcomponent	2 (1,10)	3 (23,14,11)
P06681	C2	Complement component C2	1 (2)	3 (19,15,10)
P01024	C3	Complement component C3	4 (103,106,141,147)	3 (141,105,138)
P0C0L4	C4-A	Complement component C4-A	4 (32,32,94,98)	3 (90,61,76)
P0C0L5	C4-B	Complement component C4-B	4 (32,33,95,98)	2 (90,62)
P01031	C5	Complement component C5	3 (34,23,21)	3 (68,46,70)
P13671	C6	Complement component C6	3 (20,18,4)	3 (21,14,16)
P10643	C7	Complement component C7	4 (13,9,8,6)	3 (6,11,16)
P07357	C8-A	Complement component C8 alpha chain	4 (13,11,6,7)	3 (11,8,8)
P07358	C8-B	Complement component C8 beta chain	4 (5,6,16,27)	3 (22,16,13)
P07360	C8-G	Complement component C8 gamma chain	4 (6,6,4,1)	3 (6,4,9)
P02748	C9	Complement component C9	4 (17,15,17,22)	3 (24,17,14)
P00751	FB	Complement factor B	4 (8,3,8,10)	3 (47,31,28)
P00746	FD	Complement factor D	0	3 (2,2,4)
P08603	FH	Complement factor H	2 (5,3)	3 (18,17,13)
P05156	FI	Complement factor I	1 (1)	3 (3,3,6)
P27918	FP	Complement factor P or Properdin	4 (5,5,2,2)	1 (3)
Q15485	Ficolin-2	Collagen/fibrinogen domain containing protein 2	0	2 (1,3)
O75636	Ficolin-3	Collagen/fibrinogen domain containing protein 3	1 (1)	3 (5,2,7)
O00187	MASP2	Mannan-binding lectin serine protease 2	3 (2,2,5)	1 (3)
P80108	GPLD1	Phosphatidylinositol-glycan-specific phospholipase D	0	2 (4,15)
P05155	C1INH	Plasma protease C1 inhibitor (Serpin G1)	0	3 (20,14,24)
P04004	VTN	Vitronectin or protein S	4 (10,12,10,11)	3 (14,11,13)
**COAGULATION PATHWAY**				
P01009	SERPINA1	Alpha-1-antitrypsin (AAT)	4 (4,3,6,12)	3 (37,35,47)
P08697	SERPINF2	Alpha-2-antiplasmin	4 (2,2,9,13)	3 (17,16,18)
P01008	SERPINC1	Antithrombin-III (AT-III)	4 (2,2,5,10)	3 (13,16,25)
P02749	ApoH	Beta-2-glycoprotein 1; APC inhibitor; Apolipoprotein H	2 (1,1)	3 (8,3,4)
Q96IY4	CPB2	Carboxypeptidase B2; Thrombin-activable fibrinolysis inhibitor	4 (2,1,12,10)	3 (5,3,3)
P00740	F9	Coagulation factor IX	4 (3,2,3,5)	3 (5,4,4)
P12259	F5	Coagulation factor V	4 (8,2,11,22)	3 (11,4,8)
P08709	F7	Coagulation factor VII	2 (2,3)	2 (3,2)
P00742	F10	Coagulation factor X	2 (2,2)	3 (6,6,6)
P00748	F12	Coagulation factor XII	1 (1)	2 (4,8)
P00488	F13A	Coagulation factor XIII A chain	3 (1,2,1)	2 (4,7)
P05160	F13B	Coagulation factor XIII B chain	0	3 (2,1,2)
P02671	FGA	Fibrinogen alpha chain	4 (13,12,3,7)	3 (6,6,10)
P02675	FGB	Fibrinogen beta chain	4 (13,10,1,7)	3 (6,11,14)
P02679	FGG	Fibrinogen gamma chain	3 (6,6,4)	3 (2,5,10)
P02751	FN1	Fibronectin; Binds cell surfaces and various compounds (collagen, fibrin, heparin…)	4 (60,62,15,16)	3 (29,38,24)
P68871	HBB	Hemoglobin subunit beta	4 (7,9,3,10)	3 (9,7,11)
Q9Y251	HPA	Heparanase	2 (1,3)	0
P05546	HCF2	Heparin cofactor 2 (Serpin D1)	4 (8,11,34,28)	3 (29,23,27)
P04196	HRG	Histidine-rich glycoprotein	4 (22,23,26,23)	3 (21,24,17)
Q14520	HABP2	Hyaluronan-binding protein 2	0	3 (4,3,5)
P01042	KNG1	Kininogen-1	4 (6,2,11,15)	3 (11,14,11)
P03952	KLKB1	Plasma kallikrein	4 (7,8,2,1)	3 (8,9,10)
P05154	SERPINA5	Plasma serine protease inhibitor	0	2 (3,6)
P00747	PLG	Plasminogen	4 (5,6,21,34)	3 (41,41,15)
P40197	GP5	Platelet glycoprotein V	2 (2,2)	3 (5,2,3)
Q9UK55	SERPINA10	Protein Z-dependent protease inhibitor	4 (1,1,10,9)	3 (6,5,7)
P00734	F2	Prothrombin	4 (16,18,17,25)	3 (21,17,10)
P04070	PROC	Vitamin K-dependent protein C	1 (3)	2 (3,4)
P07225	PROS1	Vitamin K-dependent protein S	3 (1,1,11)	3 (6,3,14)
P04275	VWF	von Willebrand factor	4 (18,16,19,72)	3 (52,36,9)

a Accession number, gene symbol and function from UniProtKB/Swiss-Prot database (Uniprot, 2014).

b Proteins were included if they were identified in at least two replicates of one condition with at least 2 peptides in one replicate.

This analysis rendered the identification of 372 unique *C. albicans* proteins, 371 in NS and 134 in HIS (Table S1). There were 133 proteins identified in both conditions, accounting for 36% of the total *C. albicans*-identified proteins. The Orf19.8 (an ortholog of *C. dubliniensis* CD36*)* was the only protein identified with HIS and not with NS. Noteworthy, around ~33% of the *C. albicans* proteins were identified in at least six of the seven analyzed samples. Furthermore, of the 372 *C. albicans*-identified proteins, 60 proteins presented a signal peptide (SP) at CGD. On the other hand, 214 human proteins were identified; of these, 128 were common to both conditions (60%), 43 were present only after incubation with NS and the other 43 only with HIS (Table 2 and Table S2).

### Analysis of *C. albicans* cell surface protein pattern after interaction with 10% human serum

Only those *C. albicans* identified proteins with a Gene Ontology (GO) annotation at CGD pointing either to surface localization, either to surface exposure or with the ability to induce human serum antibodies, were included in Table 1. We focused our study on these 155 *C. albicans* proteins classified into 5 categories: glycosylphosphatidylinositol (GPI)-anchored proteins; proteins involved in cell wall organization or biogenesis; cell surface proteins; plasma membrane proteins and other immunogenic proteins not present in the previous group. The groups are hierarchical and exclusive.

The first category includes 23 proteins attached to the plasma membrane or cell wall by a GPI-anchor; most are involved in biogenesis and maintenance of the *C. albicans* cell wall. All GPI-anchored proteins identified have a detected SP for entry into the secretory pathway at CGD. In the second category, there are 26 other proteins involved in cell wall organization or biogenesis. Among the 62 proteins included in the cell surface category, there are proteins located at the cell surface, although most of them do not have SP and could not reach this cell localization via the secretory pathway. Several of them have been described as “moonlighting” or multifunctional proteins. It is interesting that antibodies against a total of 52 *C. albicans*-identified proteins have been detected in human blood or serum from patients with invasive candidiasis (these immunogenic proteins are highlighted in Table 1). Many of these are abundant at the cell surface (as they were detected with more than 10 unique peptides) and were not secreted by the classical secretory pathway (not have SP), such as Eno1, Fba1, Hsp70, and 17 additional proteins.

It is also remarkable that two out of the four proteins described in *C. albicans* with the ability to fix the complement inhibitor factor H (FH) were identified. Pra1 was identified in both samples (NS and HIS), but Gpd2 was only in NS samples. Furthermore, we detected many *C. albicans* proteins described with the ability to bind plasminogen: Adh1, Eno1, Fba1, Gpd2, Pgk1, Pra1, Tdh3, Tef1, and Tsa1.

### Analysis of human serum proteins that interact with *C. albicans* cell surface relevant to *C. albicans*-host interaction

**Figure 1:**
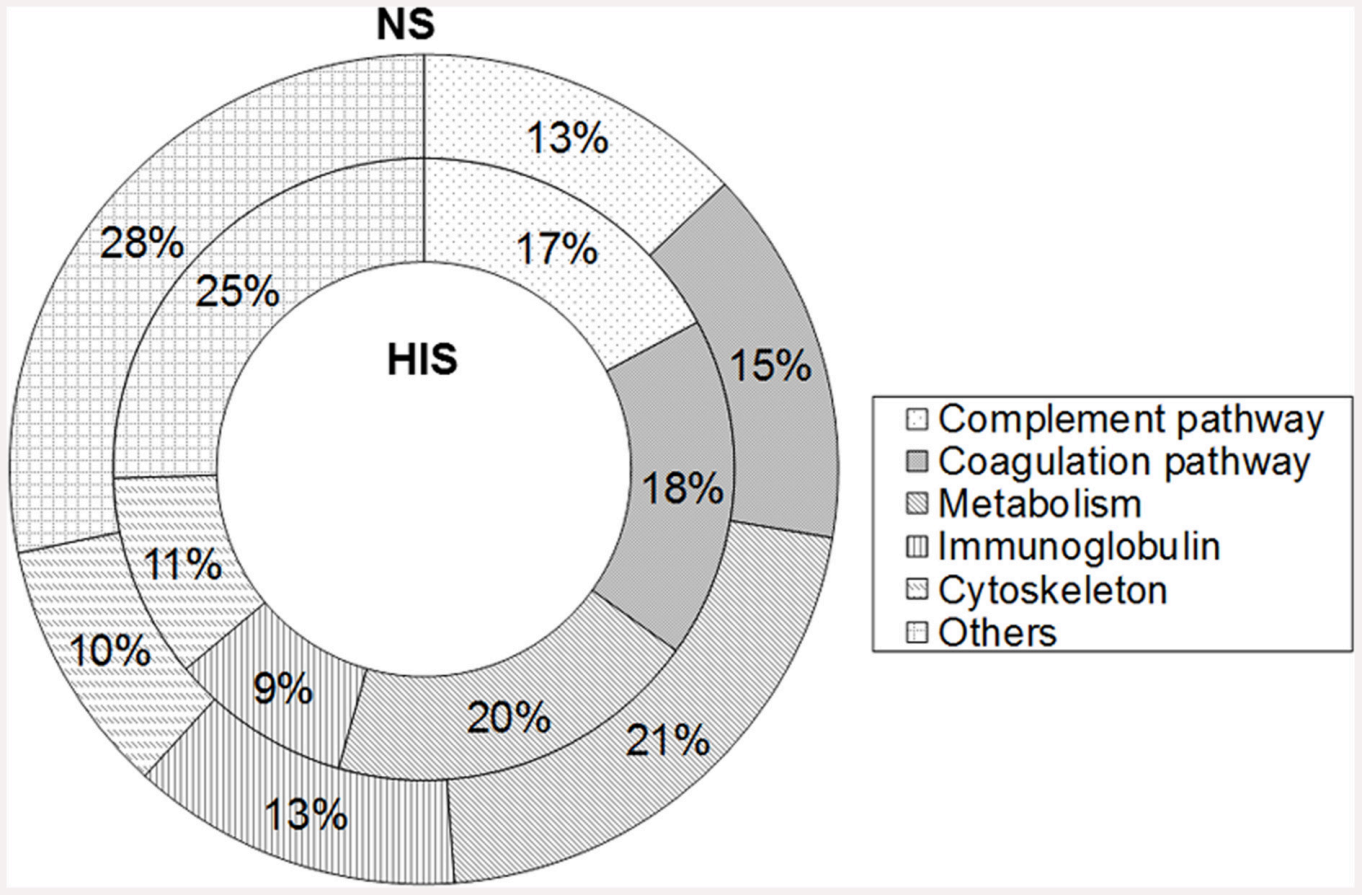
**Percentage representation of human protein groups identified in normal serum (NS) and heat inactivated serum (HIS) samples by shaving ***C. albicans*** cells**. Overall, 171 proteins were identified in both conditions and were classified into the complement pathway, coagulation pathway, metabolism, immunoglobulin, cytoskeleton, and others.

The 214 identified human proteins were classified into the following classes according to their function: complement pathway, coagulation pathway, metabolism, immunoglobulins, cytoskeleton, and others. Proteins belonging to complement and coagulation cascades are listed in Table 2 and the rest of the proteins are in Table S2. The percentages of proteins identified in each category in the two conditions tested are shown in Figure 1. The complement and coagulation pathways represent higher percentages of the total identified proteins when HIS was used and, on the other hand, the immunoglobulin group represents a higher percentage when NS was used.

Close to all components of the three complement pathways (33 proteins) were identified, most of them in the two conditions tested. However, there are some complement proteins that have been detected only in the NS sample (Collectin-11) or in the HIS sample [complement C1q subcomponent subunit A (C1qA), complement C1r-like protein, complement factor D (FD), ficolin-2, phosphatidylinositol-glycan-specific phospholipase D (GPLD1) and plasma protease C1 inhibitor (SERPING1 or C1INH)] (Table 3). The identification of many complement inhibitors such as alpha-2-macroglobulin (A2M), C1INH, FH, and factor I (FI) is also interesting. In relation to the coagulation pathway, 31 proteins were identified, with most of them also being detected in the two conditions. Although heparanase was only identified in NS sample and coagulation factor XIII B chain, hyaluronan-binding protein 2 and plasma serine protease inhibitor (SERPINA5) were only identified in the HIS one. Interestingly, antithrombin-III (SERPINC1) and plasminogen (PLG), two coagulation proteins that are relevant complement inhibitors, were identified. Focusing on human immunoglobulins IgG and IgM, they were identified in NS and HIS samples and with a similar number of peptides in both of them (Table S2). The category of metabolic proteins includes many apolipoproteins detected on the surface of *C. albicans* (Table S2).

**Table 3:** **Comparison of complement proteins identified on the cell surface of ***C. albicans*** by shaving after incubation with NS vs. HIS**.

**Complement proteins**	**Normal serum (NS)**	**Heat inactivated serum (HIS)**
Lectin pathway	7 proteins	7 proteins
	**Collectin-11**, MASP2, Ficolin-3^*^, C2^*^, C3, C4-A, C4-B	MASP2^*^, **Ficolin-2**, Ficolin-3, C2^*^, C3, C4-A, C4-B
Classical pathway	8 proteins	10 proteins
	C1qB, C1qC, C1r, C1s, C2^*^, C3, C4-A, C4-B	**C1qA**, C1qB, C1qC, C1r, **C1r-like**, C1s, C2, C3, C4-A, C4-B
Alternative pathway	3 proteins	4 proteins
	C3, FB, FP	C3, FB, **FD**, FP^*^
Membrane attack complex (MAC)	7 proteins	7 proteins
	C5, C6, C7, C8-A, C8-B, C8-G, C9	C5, C6, C7, C8-A, C8-B, C8-G, C9
Complement inhibitors	7 proteins	9 proteins
	A2M, C4BPA, C4BPB^*^, clusterin, FH, FI^*^, vitronectin	A2M, **C1INH**, C4BPA, C4BPB, clusterin, FH, FI, **GPLD1**, vitronectin

Proteins indicated in bold are only identified in one condition.

Proteins with

* are identified only in one replicate of the indicated condition.

Interestingly, 13 proteins belonging to the serpin family were identified. Serpins are a relevant group of proteins with similar structures able to inhibit proteases. Some of them had only been observed in the HIS sample, such as SERPINA5, C1INH and cortisol-binding globulin (SERPINA6) (Table 4).

**Table 4:** **Identified proteins belonging to the SERPIN family**.

**Accesion number**	**Gene name**	**Protein**	**Substrate**	**No. replicates (peptides on each replicate)**	
				**NS**	**HIS**
P01009	SERPINA1	Alpha 1-antitrypsin	Human neutrophil elastase	4 (4,3,6,12)	3 (37,35,47)
P01011	SERPINA3	Alpha 1-antichymotrypsin	Cathepsin G	2 (1,2)	3 (27,19,30)
P29622	SERPINA4	Kallistatin	Kallikrein	2 (5,10)	3 (13,19,16)
P05154	SERPINA5	Plasma serine protease inhibitor	Protein C	0	2 (3,6)
P08185	SERPINA6	Cortisol-binding globulin	Non-inhibitory; cortisol binding	0	3 (11,8,12)
P05543	SERPINA7	Thyroxine-binding globulin	Non-inhibitory; thyroxine binding	2 (2,1)	3 (6,4,14)
P01019	SERPINA8	Angiotensionogen	Non-inhibitory; Precursor of angiotensin I	4 (6,5,5,5)	3 (11,13,19)
Q9UK55	SERPINA10	Protein Z-related protease inhibitor	Factor Z and XI	4 (1,1,10,9)	3 (6,5,7)
P01008	SERPINC1	Antithrombin-III	Thrombin and factors IXa, Xa, and XIa	4 (2,2,5,10)	3 (13,16,25)
P05546	SERPIND1	Heparin cofactor II	Thrombin	4 (8,11,34,28)	3 (29,23,27)
P36955	SERPINF1	Pigment epithelium derived factor	Non-inhibitory; Potent anti-angiogenic molecule	1 (1)	3 (11,10,19)
P08697	SERPINF2	Alpha 2-antiplasmin	Plasmin and trypsin	4 (2,2,9,13)	3 (17,16,18)
P05155	SERPING1	Plasma protease C1 inhibitor	C1r, C1s, F12a, kallikrein, F11a, plasmin, MASP1, MASP2	0	3 (20,14,24)

To estimate the relative abundance of human proteins identified on *C. albicans* surface, the normalized spectral abundance factor (NSAF) (Zybailov et al., 2007) of each protein was calculated and the average NSAF values among samples were assessed. We compared these NSAF values with the ranking of the original protein abundance in human plasma using data of the dynamic range of most abundant proteins adapted from Mitchell (2010). As shown in Figure S1, the relative abundance of proteins in the plasma is different to the relative abundance at the hypha surface after interaction with human serum. For example, C3 protein of complement pathways was identified as one of the most abundant proteins on the surface of *C. albicans* in both conditions and it was not among the most abundant proteins in plasma. Also, in the apolipoprotein family, the relative abundance of some proteins is different in the plasma vs. the fungal surface. ApoB100 and ApoE are less abundant in human plasma than ApoCIII and both proteins showed higher NSAF than ApoCIII.

**Figure 2:**
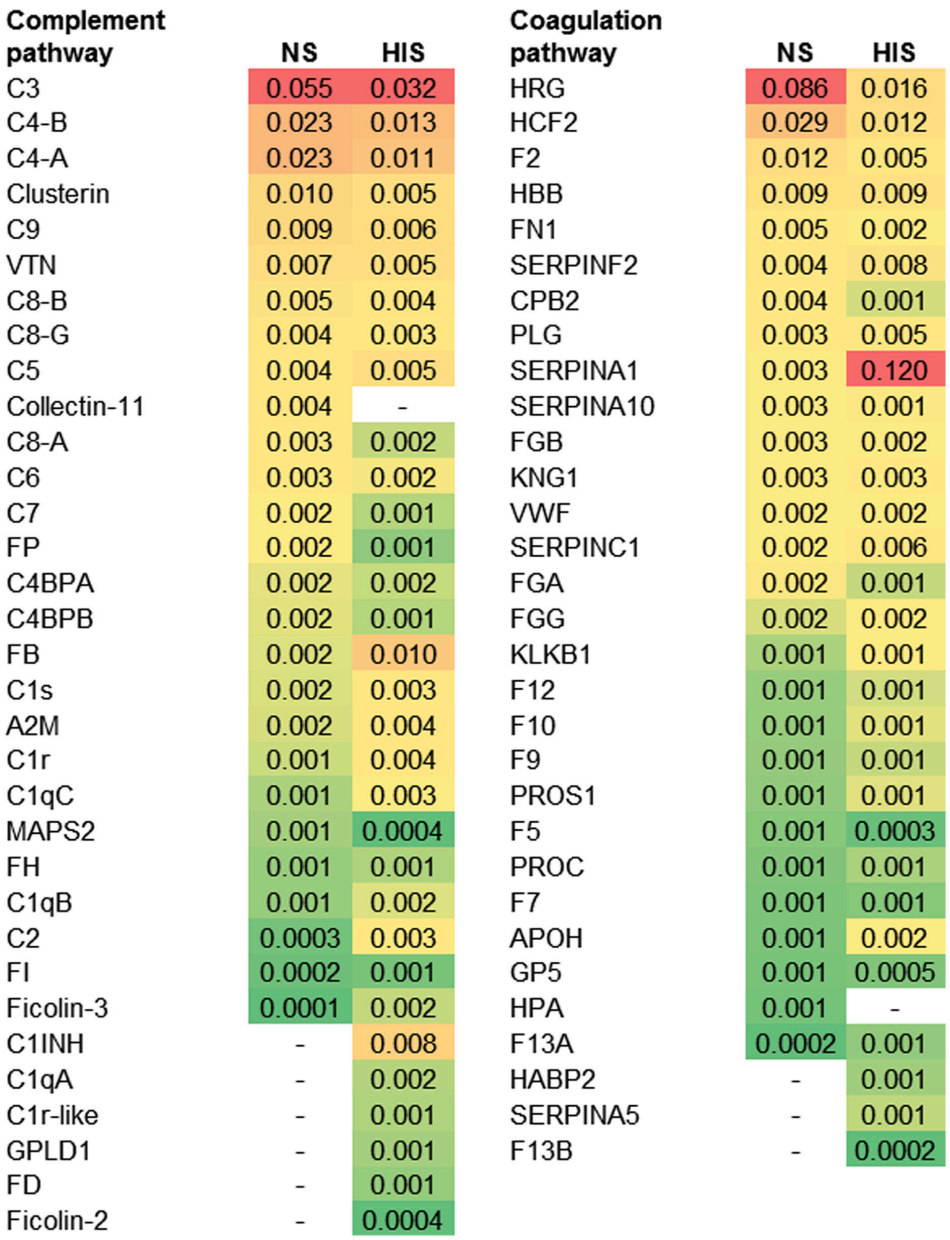
**Protein ranking of complement and coagulation proteins identified on normal serum (NS) and heat inactivated serum (HIS) samples according to the average of NSAF**. *C. albicans* was incubated with 10% human serum at 37°C (normal serum-NS or heat inactivated serum-HIS) and NSAF was calculated for proteins belonging to complement and coagulation proteins. The proteins were ordered according to their NSAF obtained in NS samples, from more to less abundant. Gradient color ranges from red (more abundant) to green (less abundant).

The abundance ranking of the proteins involved in the complement and coagulation pathways identified on *C. albicans* surface is shown in Figure 2. C3, C4-B, and C4-A had the highest NSAF results among complement proteins in both serum conditions. Complement components of Membrane Attack Complex (MAC: C6, C7, C8-A, C8-B, C8-G, and C9) had higher NSAF in NS than in HIS samples and only C5 had higher NSAF in HIS than in NS samples. In the coagulation pathway, it is notable that SERPINA1 (alpha-1-antitrypsin) is much more abundant in the HIS sample than other proteins such as HRG (histidine-rich glycoprotein), HCF2 (SERPIND1) or F2 (prothrombin), which are more abundant in the NS sample than SERPINA1.

During complement activation, C3, C4, and C5 are proteolyzed by cleavage at a specific site and small fragments are produced; these fragments are called anaphylotoxins or complement peptides, and are indicated with the letter “a” (Walport, 2001a,b). The analysis of the identified peptides belonging to: (i) anaphylotoxin regions (C3a, C4a, and C5a), (ii) released domains during proteolysis activation of complement pathways (C2b and FBa), and (iii) of the MAC-interaction regions (MACPF) is shown in Table 5. The tendency was the identification of more peptides belonging to these regions in HIS samples than in NS samples. It is remarkable that peptides from C2b and C5a were not identified in NS samples.

**Table 5:** **Analysis of peptides corresponding to cleavage fragments produced during complement activation and to contact regions in MAC formation**.

**Region**	**No. peptides**			
	**Normal Serum-NS**		**Heat Inactivated Serum-HIS**	
	**Total**	**Only in NS**	**Total**	**Only in HIS**
C2b	0	0	4	4
C3a	8	2	10	3
C4a	5	1	4	1
C5a	0	0	5	5
Ba	1	0	5	4
C6-MACPF	13	0	19	6
C7-MACPF	4	0	15	12
C8-A-MACPF	4	0	8	4
C8-B-MACPF	19	1	19	0

Identified peptides belong to the indicated released region or remain part of the full-length protein.

Gray background highlights identification in HIS condition.

### Validation of proteomic results

**Figure 3:**
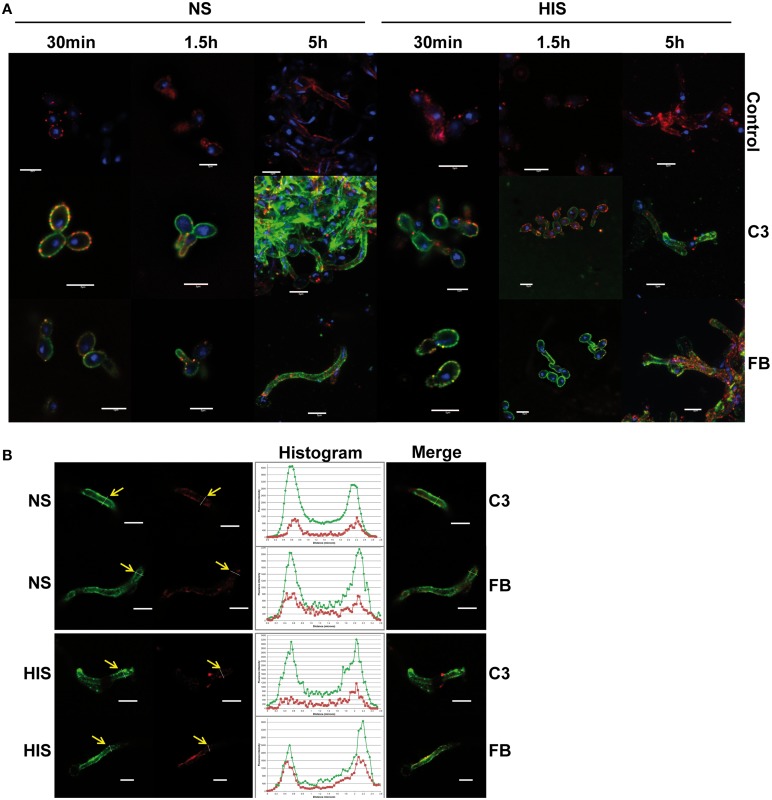
**Detection of C3 and factor B on ***C. albicans*** surface after incubation with human serum. (A)** Immunofluorescence assay to detect C3 and factor B (FB) on *C. albicans* surface incubated 30 min, 1.5 and 5 h with normal serum (NS; left panels) or heat inactivated serum (HIS; right panels). Control images showed the background of secondary antibody (anti-rabbit-A488). **(B)** Co-localization of C3 and FB with *C. albicans* plasmatic membrane (staining with PKH26) was examined by the analysis of confocal microscopy images, after 5 h of incubation with NS and HIS. Representative images are shown. Yellow arrows indicate position of transversal sections of illustrative histograms. Co-localization analysis was performed using FiJi software. Red staining of plasmatic membrane was done with PKH26, blue staining of DNA with DAPI and green staining with anti-rabbit-A488. Line in bottom right corner indicated in all figures corresponds to a 5 μm.

To validate the proteomic results, firstly the deposition level of C3 and FB on the *C. albicans* surface was analyzed by immunofluorescence microscopy and flow cytometry assays. C3 is a common component of the three complement cascades and together with A2M, are the two highest abundant complement proteins in human serum. Factor B (FB) is an abundant complement protein specific of the alternative pathway. *C. albicans* cells were incubated with NS and HIS and treated with anti-C3 and anti-FB antibodies (Figure 3). We observed the deposition of C3 and FB along the surface of all *C. albicans* cells when incubated with human serum (NS or HIS) and some areas with higher intensity in the case of C3 after 5 h of incubation with NS (Figure 3A, left panels). These differences between the fluorescence intensity of FB and C3 can be correlated with the number of peptides of these proteins in the samples at 5 h. When cells were incubated with HIS, the intensity of FB was slightly higher than in the case of NS incubation (it is well observed in short incubation times) (Figure 3A, right panels). To achieve flow cytometry assays, the incubation time of *C. albicans* cells with human serum was decreased to avoid the formation of large filaments. Within 30 min of incubation, the deposition of C3 was higher among the population in NS samples than in HIS (Figure S3). The intensity of FB was lower than that of C3, as expected, and less homogenous in both samples (NS and HIS) (upper panels). Mean fluorescence intensity (MFI) of two independent experiments were collected and showed that, in all cases, the MFI for C3 and FB was higher when cells were incubated with NS than with HIS at short times, although the differences are not significant in the case of FB (bottom panel).

All of these data together showed that C3 and FB deposition was clearly observed at short incubation times, and C3 enhanced its accumulation on the surface with longer incubation time and more dramatically when the incubation was with NS. However, FB accumulation on *C. albicans* surface did not appear to increase with time, maybe because it can be masked by the rest of the complement proteins (Figure 3A).

Furthermore, to address whether C3 and FB are close to the cytoplasmic membrane, fluorescence co-localization was evaluated with confocal microscopy images by the analysis of transversal sections across cells (Figure 3B). As observed, both C3 and FB proteins are localized around the plasmatic membrane, staining when *C. albicans* cells were incubated with NS and also with HIS.

Afterwards, in order to evaluate the deposition of human IgGs on *C. albicans* surface after 5 h of incubation with 10% human serum, IgGs were detected in NS and HIS samples, with higher intensity on the yeast surface than on the hyphal surface in both samples (Figure S2).

## Discussion

Our work shows, for the first time to our knowledge, the identification by a shaving approach of 147 *C. albicans* surface proteins and 214 human serum proteins attached to the surface of the fungus in the same report. The more relevant *C. albicans* proteins identified are GPI-anchored proteins and proteins involved in cell wall organization or biogenesis. The most relevant human proteins identified belong to complement and coagulation pathways. Indeed, all main human complement proteins were identified on the surface of *C. albicans*. The identified proteins were reproducible among the experiments and would represent the physiological mode of action of the immune system against *C. albicans* or components of the immune system used for *C. albicans* to evade, disturb or use on its own purpose the host defenses.

### Analysis of *C. albicans* surface proteins

With the objective of unraveling the protein surface pattern of *C. albicans* hyphal cells, we analyzed our data in comparison to the data obtained by our group using the same methodology of cell shaving over yeast and hyphal cells (growth also in Lee's medium, but without human serum) (Gil-Bona et al., 2015). This analysis rendered 304 proteins identified in all *C. albicans* assays (yeasts and hyphae with and without human serum) that we consider the *C. albicans* common core of cell surface proteins and not morphotype specific (Table S3). Also, a hyphal protein pattern of 55 proteins identified on *C. albicans* hypha forms, but not identified in *C. albicans* yeasts, was obtained. Among them, Als3, Hyr1, and Sod5 were associated with hyphae in different works (Heilmann et al., 2011; Sosinska et al., 2011; Sudbery, 2011). They are proteins that are expected to be detected, indicating that the other 52 proteins are at least more abundant or accessible in hypha than in yeast cells. Another example is Ece1, which is a hyphal-specific protein with unknown function (Fan et al., 2013) (Table S3).

Other proteins were associated with hyphae in some works and described as morphotype-independent in others, for example, Phr1 and Rbt1 (Heilmann et al., 2011; Ragni et al., 2011; Monniot et al., 2013). We identified both proteins in all shaving *C. albicans* experiments. Furthermore, Phr2 (a homolog of Phr1) was also identified in all conditions. The differences observed at the level of detection of Phr1 and Phr2 among the samples were possibly related to the optimum pH for expression of each one (Sosinska et al., 2011; Dühring et al., 2015) (Table S1). On the other hand, Rhd3 and Ywp1 were reported as yeast-specific and were found to be part of the common core pattern in our study (Heilmann et al., 2011). A relevant protein found in *C. albicans* common core pattern is Msb2; this stabilizes the fungal cell wall and inactivates human antimicrobial peptides (Dühring et al., 2015).

It is interesting to know that GPI-anchored proteins identified in this work are part of the common core of *C. albicans* surface proteins or of the hyphal-form specific ones. There are 17 proteins belonging to the common core in our analysis, and also Ece1 and Sod5, which are part of the hyphal-specific group, whose genes were up-regulated under blood growth (Fradin et al., 2003).

Remarkably, 12 proteins were only identified on the *C. albicans* hyphal surface when it was grown with 10% human serum and we highlighted Pra1, Sap5, Tef1, and 5 more Orfs with unknown function (Table S3). In this work, two members of the Sap family, Sap9 and Sap5, were identified. Sap9 is part of *C. albicans* common core while Sap5 shows up in hyphae induced with the human serum group. Both genes were up-regulated in *C. albicans* biofilm associated with bloodstream infections (Joo et al., 2013). Ten out of these twelve proteins are identified for the first time on the surface of *C. albicans*; Pra1 and Tef1 have been previously identified in the cell wall of *C. albicans*.

Many proteins described as “moonlighting” or multifunctional proteins in *C. albicans* were identified in this work (Nombela et al., 2006; Chaffin, 2008) (indicated with ^*^ in Table S1). Among them, Gpd2 has been described as a virulence factor on microorganism surface because it mediates complement evasion; meanwhile, there is a glycerol 3-phosphate-dehydrogenase in the cytoplasm that is involved in the intracellular accumulation of glycerol to control osmotic pressure (Luo et al., 2013). Tsa1 is involved in oxidative stress response in the cytoplasm and in hyphal cell wall biogenesis (Urban et al., 2005). Tdh3 is a glycolytic enzyme (glyceraldehyde-3-phosphate dehydrogenase) in the cytoplasm and able to attach fibronectin and laminin at the cell surface (Gozalbo et al., 1998).

Regarding *C. albicans* proteins that interact with complement inhibitor proteins (FH and PLG), the expression of Gpd2 and Pra1 is more prominent at the hyphal tip (Zipfel et al., 2011; Luo et al., 2013). Pra1 is also able to bind C4BP and it is also known that the hyphal tip is a prominent binding site for C4BP (Meri et al., 2004; Luo et al., 2011). For these reasons and because it facilitates host tissue penetration, the hyphal tip is considered an important factor of pathogenesis. We identified these two proteins on the *C. albicans* surface grown with 10% human serum that activate hyphal growth. Focusing on the mechanism to evade complement activation mediated by plasminogen coated surfaces, as plasminogen is an important complement cascade inhibitor in the fluid phase and on the human surface (Barthel et al., 2012), the identification of nine out of the eleven *C. albicans* proteins with the ability to bind PLG (Adh1, Eno1, Fba1, Gpd2, Pgk1, Pra1, Tdh3, Tef1, and Tsa1) is another highlight of this study (Crowe et al., 2003; Jong et al., 2003; Barthel et al., 2012).

### Human serum proteins with the ability to interact with *C. albicans* surface

We detected human plasma proteins from different abundance strata on *C. albicans* surface; most of them are from the most abundant proteins or from intermediate stratum (Anderson and Anderson, 2002; Mitchell, 2010). Due to the heat treatment of HIS serum, FB and C2 become non-functional; in this situation, other human serum proteins could interact further with the surface of *C. albicans*. Remarkably, *C. albicans* surface could be less accessible to trypsin digestion when it was grown with 10% HIS as the number of coated of human proteins is greater. This could explain the observation of higher sum of peptide-spectrum match (PSMs) of human serum proteins in the HIS condition in comparison with the NS condition. In any case, it is not possible to discard that some proteins differentially observed in HIS are a result of changes in their binding properties due to protein denaturation.

Albumin is the most abundant protein in human plasma with close to two orders of magnitude more than the rest of proteins (Mitchell, 2010), but it was not detected as the highest abundant protein on *C. albicans* surface incubated with human serum (either NS or HIS) (Figure S1). Also, there are some proteins only detected on *C. albicans* surface in HIS samples that have intermediate abundance in human plasma such as C1INH, C1qA, and RBP4. Serotransferrin and fibrinogen (FGG, FGA and FGB) were more abundant in NS condition and in the other side apolipoprotein A-I and transthyretin in HIS condition. All of these data indicate that the interaction between human proteins and *C. albicans* surface did not respond solely to their protein abundance in human serum. The study of C3 and FB deposition on *C. albicans* surface after incubation with 10% human serum was carried out by immunofluorescence with specific antibodies. The clearly deposition of C3 and FB was observed at short times of interaction in Figure 3A.

**Figure 4:**
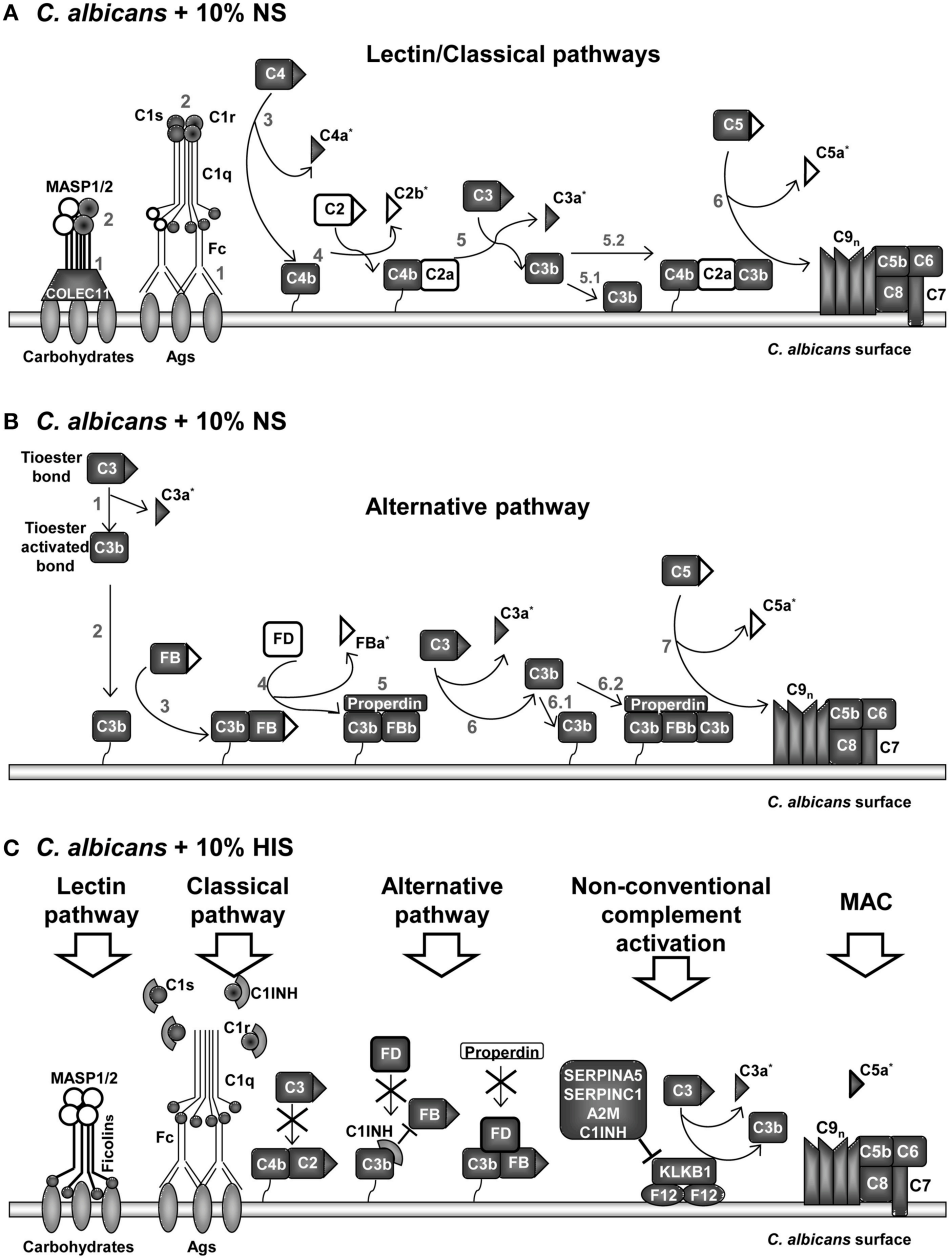
**Model of interactions among proteins of the complement pathways identified on ***C. albicans*** surface after incubation with human serum**. Proteins identified in normal serum (NS) samples in **(A,B)**. Proteins belonging to lectin and classical pathways are represented in **(A)** and to alternative pathway in **(B)**. Proteins mainly identified on heat inactivated serum (HIS) in **(C)**. Identified proteins on samples are indicated with gray background and not-identified in white. Complement fragments released during complement activation are indicated with triangles. Peptides belonging to complement fragments that can be released or be part of a full-length protein are indicated with ^*^. Numbers in **(A,B)** correspond to sequential steps during complement pathways activation. **(A)** Collectin-11 was only identified in NS samples. MASP2 was identified in NS and MASP1 was not identified in any sample. Fc (constant region of Ig) of IgGs or IgMs start classical complement pathway. **(B)** Properdin was identified in NS and Factor D (FD) was not. **(C)** Ficolin-2, Ficolin-3, C1qA, C1INH, and FD were identified only in HIS samples. C1INH dissociates the C1qrs complex, blocks alternative C3 convertase and inhibits C3 activation by kallikrein. C2 was identified in HIS, including 4 peptides of C2b. Five peptides belonging to FBa were identified in HIS condition. Signally, peptides belonging to C5a fragment were only identified in HIS samples.

A graphical summary of the identified proteins at *C. albicans* surface from the different complement pathways in NS and HIS conditions is presented in Figure 4. The enrichment on the surface of *C. albicans* in proteins belonging to the lectin, classical and alternative complement pathways is visibly observed in normal conditions (NS); almost all of the main proteins of complement pathways were identified (Figures 4A,B). Peptides belonging to anaphylotoxin region are less frequently identified in NS than in HIS condition; this could be related to that C2b, C3a, FBa, and C5a fragments were released to the medium under this condition (Table 5). Furthermore, two peptides (CCEDGMR and CCEDGMRENPMR), which are part of the LRK26 peptide derived from C3a and with antimicrobial effects on *C. albicans* surface (Sonesson et al., 2007), were only identified in NS. Peptides belonging to the C5a region were only identified in HIS samples, just as more peptides belonging to the MACPF region of C6, C7, and C8-A. Furthermore, lower NSAF for all components of MAC, except C5, were obtained with HIS (Figure 2), which could be correlated with the enhanced identification of C5a peptides in this condition. In contrast, no identification of peptides belonging of C5a region and higher NSAF for the rest of components of MAC in NS samples may indicate MAC formation. MACPF-domains would not be accessible to trypsin digestion because they are points of interaction for MAC formation and would be protected in this condition. Furthermore, we identified clusterin and vitronectin (VTN) with higher NSAF in NS condition and both are able to interact with MAC in plasma, and thus prevent the cytolysis of host cells (Chauhan and Moore, 2006). Further experiments are required to determine the formation and the functionality of MAC on *C. albicans* surface.

In the same line of thought, in NS samples, properdin (FP) and not factor D (FD) were identified, while the opposite happened with HIS samples (Figure 4B vs. Figure 4C). The results are in agreement with a functional alternative complement pathway in NS condition on the surface of *C. albicans*. On the other hand, in HIS samples, FD could still be attached to the C3bB complex and we suppose that C3 convertase is not formed. In this situation, FP could not stabilize the complex and would be not identified on HIS samples. C1INH is able to bind to immobilized C3b and block progression of the complement cascade (Jiang et al., 2001). Serpin role of C1INH produces dissociation of C1qrs complex and the identification of these proteins in the HIS condition was enhanced, possibly for this reason. C3 and C4 (C4-A and C4-B) were identified with less NSAF and C2 and FB with higher NSAF in HIS samples (Figure 4C). In a current study about *Streptococcus pyogenes* interaction with human plasma, C2, C1qA, and FD were not detected on the surface of the microorganism (Sjöholm et al., 2014). In our experiments, when *C. albicans* cells were incubated with HIS, we detected not only these proteins, but also C1INH, and the enrichment of FB, C1qB, and C1qC. This is an interesting observation that may be due to the absence of functional convertases; FB and C2 might be more accessible to trypsin digestion. We assume that the unsuitable formation of complement convertases, or at least in a less extended manner, is responsible for the enhanced peptide identification of C2, FB, FD, C5, and the C1qrs complex in HIS samples.

Coagulation and complement pathways are highly connected, with many cross-talks (Kazatchkine and Jouvin, 1984; Amara et al., 2008, 2010; Peerschke et al., 2008; Diamond, 2013; Verschoor and Langer, 2013). Many complement inhibitors were identified on the surface of *C. albicans* and in general more abundant in HIS samples. The innate immune system strives to respond and activates complement cascades by non-conventional mechanisms (thrombin (F2), PLG, Factor XII (F12), kallikrein (KLKB1), ficolin-2 and -3) and bypass locking points in absence of functional FB and C2 (Nielsen et al., 1996; Rittirsch et al., 2008) (Figures 2, 4C). In HIS samples, KLKB1 inhibitors were enriched, such as SERPINA5, SERPINC1, A2M, and C1INH (Gadjeva et al., 1998; Paréj et al., 2013).

A relevant group of proteins is serpin family (serine protease inhibitors), which are broadly distributed among eukaryote organisms. Serpins are able to inhibit proteases by a specific mechanism where covalent complexes are formed with target proteases (Law et al., 2006). Since serpin has to be cleaved to inhibit the target substrates, inhibition also consumes the serpin; serpins are therefore irreversible enzyme inhibitors. For these reasons, the identification of many serpins on *C. albicans* surface and more interestingly in HIS samples is an interesting observation because they may be inactivating their substrates attached to the surface of *C. albicans* or themselves coating *C. albicans* surface in the sense to avoid interactions between their substrates and *C. albicans* (Table 4).

Interestingly, the apolipoproteins classified in the metabolism group are involved in lipid transport and also play a relevant role in host defense as part of the innate immune system (Grunfeld and Feingold, 2008). Furthermore, C*. albicans* grows better in lipoprotein-depleted plasma due to two factors: greater availability of lipids and reduced neutralizing candidacidal factor (Vonk et al., 2004). ApoE has a relevant role in systemic candidiasis infection and showed higher NSAF in NS samples (Figure S1). ApoE-/- mice showed an increased mortality in comparison with wild type (85 vs. 52%) (Vonk et al., 2004), and also an impaired immune response in *Klebsiella pneumonia* infection (de Bont et al., 2000).

In summary, the mechanisms developed by *C. albicans* to evade, interfere or use for its benefit host defenses are very wide and part of its pathogenic evolution. The analysis of *C. albicans* surface after incubation with human serum to obtain a partial breakdown of the immunological crosstalk between *C. albicans* and human serum proteins is a relevant target of study. *C. albicans* mimic human immune evasion model using many surface proteins to fix plasminogen and complement inhibitors (FH and C4BP) to protect against complement activation. Furthermore, the identification of Pra1, Sap5, Tef1, and 9 more proteins only identified in *C. albicans* hyphal surface induced with 10% human serum and not in other published conditions with an equivalent approach is a relevant highlight of this analysis.

## Author contributions

EM designed and performed the experiments, analysis of results, and writing of the manuscript. CP designed and performed the experiments. CH performed the experiments. MH performed the experiments and analysis of results. CN research support. LM designed the experiments, analysis of results and critically revised the manuscript. CG designed the experiments, analysis of results, and critically revised the manuscript.

### Conflict of interest statement

The authors declare that the research was conducted in the absence of any commercial or financial relationships that could be construed as a potential conflict of interest.
