# Supplementary material for: Candida albicans Shaving to Profile Human Serum Proteins on Hyphal Surface
Source: Front Microbiol. 2015 Dec 8;6:1343. doi: 10.3389/fmicb.2015.01343 (PMC4672057; doi:10.3389/fmicb.2015.01343)
Supplement: Supplementary file 1 [file Image1.PDF]

Human plasma proteins	Relative concentration	NS	HIS
Serum albumin (ALB)	10000	0.0303	0.0273
Apolipoprotein A-I (ApoAI)	800	0.0258	0.0817
Serotransferrin (TF)	700	0.0074	0.0037
Alpha-1-antitrypsin (SERPINA1)	700	0.0028	0.1203
Haptoglobin (HP)	600	0.0105	0.0090
Transthyretin (TTR)	400	0.0063	0.0110
Fibrinogen gamma chain (FGG)	200	0.0019	0.0015
Fibrinogen alpha chain (FGA)	200	0.0023	0.0009
Fibrinogen beta chain (FGB)	200	0.0027	0.0017
Apolipoprotein C-III (ApoCIII)	100	0.0039	0.0051
Alpha-2-macroglobulin (A2M)	90	0.0015	0.0035
Complement C3	70	0.0550	0.0325
Antithrombin-III (SERPINC1)	60	0.0024	0.0057
Complement factor H (FH)	40	0.0008	0.0014
Complement factor B (FB)	40	0.0016	0.0099
Ceruloplasmin (CP)	30	0.0017	0.0029
Complement component C9	20	0.0094	0.0056
Plasma protease C1 inhibitor (C1INH)	20	-	0.0083
Complement C1q subcomponent, C chain	20	0.0010	0.0026
Complement C1q subcomponent, B chain	20	0.0007	0.0020
Complement C1q subcomponent, A chain	20	-	0.0015
Prothrombin (F2)	15	0.0120	0.0053
Plasma retinol-binding protein (RBP4)	15	-	0.0022
Complement C4-A	15	0.0228	0.0113
Complement C4-B	15	0.0230	0.0130
Apolipoprotein B-100 (ApoB100)	15	0.0225	0.0176
Alpha-2-antiplasmin (SERPINF2)	15	0.0044	0.0080
Plasminogen (PLG)	10	0.0034	0.0055
Apolipoprotein E (ApoE)	10	0.0124	0.0098
Complement component C8 gamma	9	0.0044	0.0031
Complement component C8 alpha chain	9	0.0035	0.0017
Complement component C8 beta chain	9	0.0051	0.0037
Complement factor I (FI)	8	0.0002	0.0007
Complement component C7	7	0.0020	0.0015
Complement component C6	6	0.0029	0.0023
Thyroxine-binding globulin (SERPINA7)	6	0.0004	0.0029
Coagulation factor XII (F12)	5	0.0010	0.0010
Complement C5	5	0.0037	0.0054
Vitamin K-dependent protein S (PROS1)	4	0.0009	0.0011
Apolipoprotein(a) (Apo(a))	4	0.0003	0.0003
Coagulation factor X (F10)	3	0.0010	0.0011
Complement C2	2	0.0003	0.0029
Beta-2-glycoprotein 1 (ApoH)	1	0.0006	0.0020

Figure S1. Protein ranking according to the averaged normalized spectral abundance factor (NSAF) in comparison with original abundance on human plasma. *C. albicans* cells were incubated with 10% human serum at 37ºC for 5h (normal serum-NS or heat inactivated serum-HIS). For more abundant proteins in human plasma that were identified on samples (NS or HIS), the Normalized Spectral Abundance Factor (NSAF) was calculated and compared with the original abundance in human plasma. The relative concentration of human plasma proteins was adapted from Mitchell et al., 2010; the relative concentrations shown are the data x10^5. NSAF ranges from more abundant (red) to less abundant (green) proteins.
