# Supplementary material for: Candida albicans Shaving to Profile Human Serum Proteins on Hyphal Surface
Source: Front Microbiol. 2015 Dec 8;6:1343. doi: 10.3389/fmicb.2015.01343 (PMC4672057; doi:10.3389/fmicb.2015.01343)
Supplement: Supplementary file 2 [file Image2.PDF]

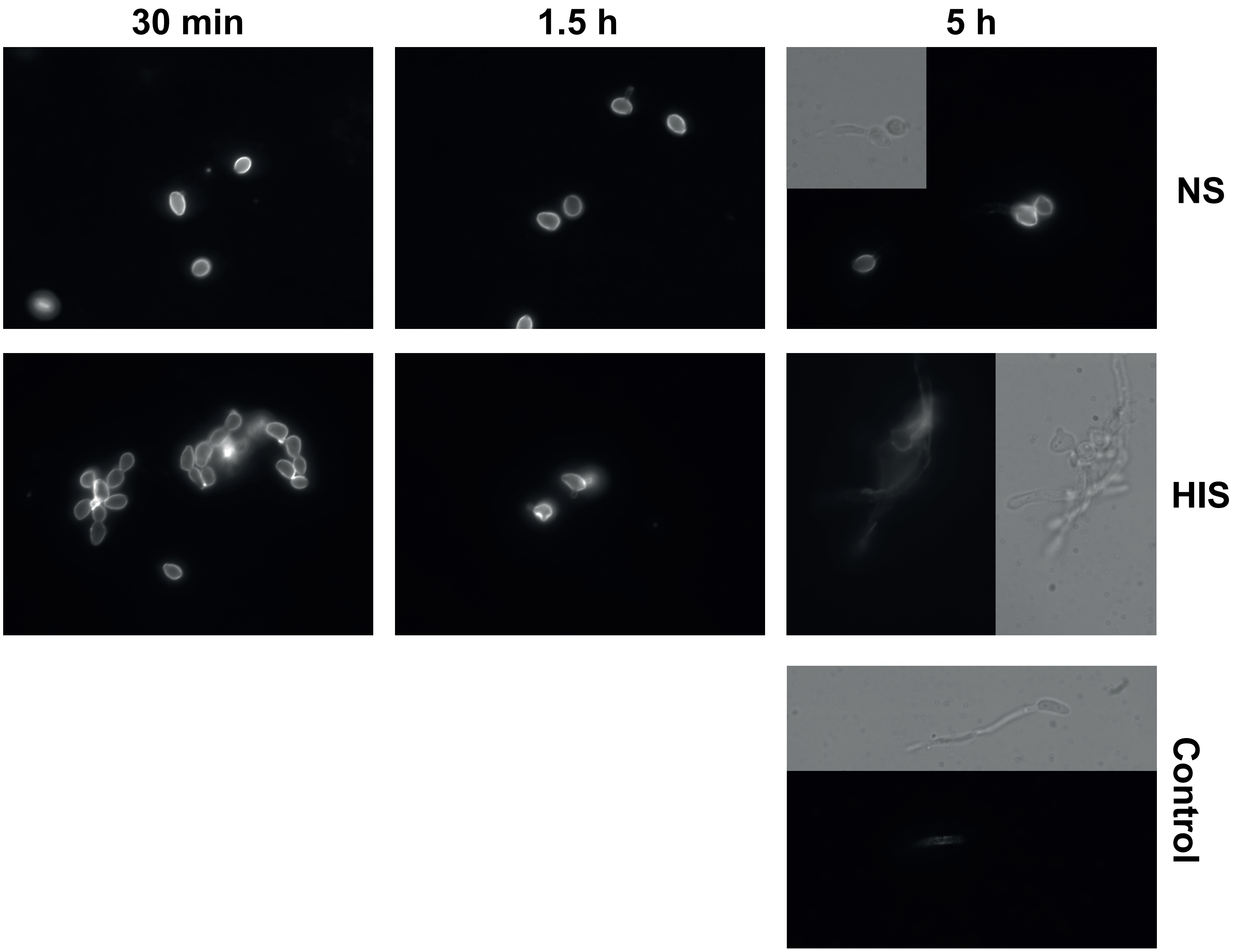

Figure S2. Detection of IgGs human on *C. albicans* surface after interaction with human serum. To identify IgGs human levels on *C. albicans* surface after incubation with 10% human serum, we performed an immunofluorescence assay with anti-Human IgG PE conjugated (eBioscience) following the manufacturer´s instructions. *C. albicans* was incubated for 30 min, 1.5 h or 5 h with 10% human serum at 37 ºC in order to evaluate IgG human levels on its surface. Upper panel with normal serum (NS), middle panel with heat inactivated serum (HIS) and bottom panel without human serum, as a control background. Panels overlaid in 5 h incubations are phase contrast images to observe whole cells.
