# Supplementary material for: Candida albicans Shaving to Profile Human Serum Proteins on Hyphal Surface
Source: Front Microbiol. 2015 Dec 8;6:1343. doi: 10.3389/fmicb.2015.01343 (PMC4672057; doi:10.3389/fmicb.2015.01343)
Supplement: Supplementary file 3 [file Image3.PDF]

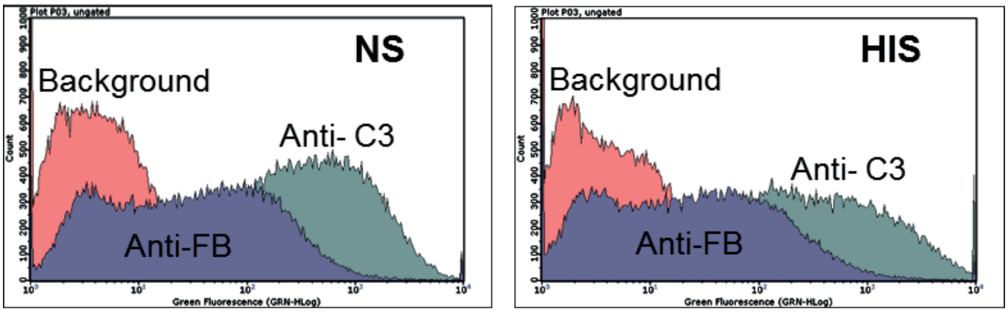

***C. albicans* + 10% serum**		MFI ± SD
**NS**	**Background**	4.8 ± 1
**NS**	**Anti-C3**	290.8 ± 30
**NS**	**Anti-FB**	33.4 ± 6
**HIS**	**Background**	4.5 ± 1
**HIS**	**Anti-C3**	156.3 ± 61
**HIS**	**Anti-FB**	19.3 ± 7

Figure S3. Detection of C3 and factor B on *C. albicans* surface after incubation with human serum. Fluorescent flow cytometry analysis of C3 and FB after incubation *C. albicans* with NS (left panel) or HIS (right panel) during 30 min. Graph below histograms corresponds to an average of mean fluorescence intensity (MFI) between two independent experiments. Background in all cases corresponds to cells incubated only with secondary antibody and without primary antibody. Secondary antibody is anti-rabbit IgG-Alexa488.
