# Supplementary material for: Candida albicans Shaving to Profile Human Serum Proteins on Hyphal Surface
Source: Front Microbiol. 2015 Dec 8;6:1343. doi: 10.3389/fmicb.2015.01343 (PMC4672057; doi:10.3389/fmicb.2015.01343)
Supplement: Supplementary file 4 [file Table1.DOC]

**Table S1.** *C. albicans* proteins identified by shaving after incubation with 10% human serum.

| Identifier^a)^ | Protein name^a)^ | Description^a)^ | No. replicates  (peptides on each replicate)^b)^  **NS HIS** | | Signal peptide^a)^ |
| --- | --- | --- | --- | --- | --- |
| Orf19.2699 | Abp1 | Protein similar to *S. cerevisiae* Abp1p, which is an actin-binding protein of the cortical actin cytoskeleton; caspofungin induced | 4 (9,6,5,1) | 2 (2,2) |  |
| Orf19.7043 | Acb1 | Protein similar to a region of acyl-coenzyme-A-binding protein; amphotericin B repressed | 4 (2,2,2,2) | 2 (1,1) |  |
| Orf19.7466 | Acc1 | Putative acetyl-conenzyme-A carboxylases; amphotericin B repressed; caspofungin repressed | 4 (8,5,9,3) | 0 |  |
| Orf19.1046 | Acs2 | Acetyl-CoA synthetase; antigenic during human and murine infection | 4 (8,7,7,3) | 1 (1) |  |
| Orf19.5007 | Act1 | Actin; localizes to polarized growth site in budding and hyphal cells | 4 (10,8,15,12) | 3 (9,7,5) |  |
| Orf19.4827 | Ade12 | Adenylosuccinate synthase | 2 (1,2) | 0 |  |
| Orf19.492 | Ade17 | 5-Aminoimidazole-4-carboxamide ribotide transformylase, enzyme of adenine biosynthesis; antigenic in human | 4 (5,4,6,2) | 1 (2) |  |
| Orf19.3997 | Adh1 * | Alcohol dehydrogenase; binds human plasminogen | 4 (15,15,17,17) | 3 (11,16,3) |  |
| Orf19.5113 | Adh2 | Alcohol dehydrogenase; fungal-specific (no human or murine homolog) | 3 (3,3,3) | 1 (3) |  |
| Orf19.3391 | Adk1 | Putative adenylate kinase | 4 (3,2,1,1) | 1 (1) |  |
| Orf19.5591 | Ado1 | Adenosine kinase; ketoconazole-induced | 4 (4,4,5,1) | 2 (1,2) |  |
| Orf19.5013 | Agm1 | Phosphoacetylglucosamine mutase (N-acetylglucosamine-phosphate mutase) | 2 (2,1) | 1 (2) |  |
| Orf19.2762 | Ahp1 * | Putative alkyl hydroperoxide reductase, immunogenic in mouse; fluconazole-induced; amphotericin B, caspofungin, alkaline downregulated; induced in core stress response | 4 (5,5,5,5) | 3 (5,7,2) |  |
| Orf19.5746 | Ala1 | Alanyl-tRNA synthetase | 2 (2,1) | 0 |  |
| Orf19.5741 | Als1 | Adhesion, band hyphal base | 4 (4,3,3,2) | 0 | Yes |
| Orf19.1816 | Als3 | ALS family cell wall adhesion | 4 (9,9,9,7) | 3 (5,7,2) | Yes |
| Orf19.3426 | Anb1 | Translation initation factor eIF-5A | 4 (4,3,2,2) | 2 (2,2) |  |
| Orf19.5197 | Ape2 | Neutral arginine-, alanine-, leucine-specific metallo-aminopeptidase | 3 (1,1,6) | 2 (1,1) | Yes |
| Orf19.5964 | Arf2 | Putative ADP-ribosylation factor; mutation confers hypersensitivity to Brefeldin A | 3 (3,4,2) | 0 |  |
| Orf19.7469 | Arg1 | Argininosuccinate synthase | 4 (3,2,8,2) | 1 (2) |  |
| Orf19.4704 | Aro1 | Putative pentafunctional arom enzyme; fungal-specific (no human or murine homolog) | 2 (1,2) | 0 |  |
| Orf19.4060 | Aro4 | 3-deoxy-D-arabinoheptulosonate-7-phosphate synthase; aromatic amino acid biosynthesis | 3 (3,1,4) | 1 (1) |  |
| Orf19.2098 | Aro8 | Aromatic transaminase of the Ehrlich fusel oil pathway of aromatic alcohol biosynthesis | 3 (3,1,2) | 0 |  |
| Orf19.6906 | Asc1 | 40S ribosomal subunit similar to G-beta subunits; required for virulence in mice | 4 (6,6,7,5) | 2 (4,4) |  |
| Orf19.198 | Asn1 | Putative asparagines synthetase; soluble protein in hyphae | 4 (2,2,1,2) | 2 (1,1) |  |
| Orf19.2344 | Asr1 | Putative heat shock protein; transcription regulated by cAMP, osmotic stress, ciclopirox olamine, ketoconazole | 2 (2,1) | 0 |  |
| Orf19.842 | Asr3 | Gene regulated by cAMP and by osmotic stress | 4 (1,1,2,2) | 0 |  |
| Orf19.6854 | Atp1 * | ATP synthase alpha subunit; antigenic in human/mouse; at hyphal surface | 3 (1,2,1) | 1 (1) |  |
| Orf19.5653 | Atp2 | F1 beta subunit of F1F0 ATPase complex; antigenic in human, mouse; induced by ciclopiox olamine; caspofungin repressed | 2 (3,3) | 2 (1,1) |  |
| Orf19.6994 | Bat22 | Putative branched chain amino acid aminotransferase | 2 (2,1) | 0 |  |
| Orf19.2014 | Bcy1 | Protein kinase A regulatory subunit | 3 (2,3,2) | 0 |  |
| Orf19.4560 | Bfr1 | Protein involved in the maintenance of normal ploidy | 3 (3,1,4) | 1 (2) |  |
| Orf19.4565 | Bgl2 | 1,3-beta-glucosyltransferase cell wall enzyme | 4 (4,3,5,3) | 1 (3) | Yes |
| Orf19.3014 | Bmh1 * | Sole 14-3-3 protein in *C. albicans*; role in hyphal growth | 4 (8,8,11,7) | 3 (5,7,1) |  |
| Orf19.1699 | Bzz1 | Protein similar to *S. cerevisiae* Bzz1p, which is an SH3 domain protein involved in the regulation of actin polymerization | 2 (2,3) | 0 |  |
| Orf19.7382 | Cam1 | Putative translation elongation factor eEF1 gamma | 4 (7,5,10,1) | 2 (3,1) |  |
| Orf19.2651 | Cam1-1 | Putative translation elongation factor | 4 (6,4,8,1) | 0 |  |
| Orf19.5641 | Car2 | Ornithine aminotransferase; role in arginine metabolism; mutation confers hypersensitivity to toxic ergosterol analog and amphotericin B | 4 (2,2,2,2) | 0 |  |
| Orf19.3206 | Cct7 | Cytosolic chaperonin Cct ring complex | 2 (1,2) | 0 |  |
| Orf19.3013 | Cdc12 | Septin; essential for viability | 3 (5,2,2) | 1 (1) |  |
| Orf19.3575 | Cdc19 | Pyruvate kinase; on yeast cell surface | 4 (10,14,20,11) | 3 (9,8,3) |  |
| Orf19.1055 | Cdc3 | Septin; essential for viability; down-regulation associated with azole resistance | 4 (4,3,5,2) | 1 (1) |  |
| Orf19.390 | Cdc42 | Rho-type GTPase; required for budding and maintenance of hyphal growth; GGTase I geranylgeranylated; misexpression blocks hyphal growth, causes avirulence in mouse IV infection | 2 (1,2) | 0 |  |
| Orf19.2340 | Cdc48 * | Described microsomal ATPase | 4 (8,7,7,4) | 2 (2,4) |  |
| Orf19.2560 | Cdc60 | Cytosolic leucyl tRNA synthetase; interact with benzoxaborole antifungals | 3 (4,1,5) | 0 |  |
| Orf19.4152 | Cef3 | Translation elongation factor 3; antigenic in humans; possibly essential | 4 (24,19,28,13) | 3 (6,9,1) |  |
| Orf19.3496 | Chc1 | Ortolog(s) have role in endocytosis and cytoplasm localiztion | 3 (4,1,1) | 0 |  |
| Orf19.3895 | Cht2 | GPI-linked chitinase | 4 (7,5,8,3) | 3 (1,5,2) | Yes |
| Orf19.7586 | Cht3 | Major chitinase | 1 (1) | 2 (2,1) | Yes |
| Orf19.113 | Cip1 | Possible oxidoreductase | 3 (2,1,5) | 0 | Yes |
| Orf19.4413 | Cmd1 | Calmodulin | 2 (3,2) | 1 (1) |  |
| Orf19.953.1 | Cof1 | Cofilin | 4 (3,2,4,1) | 2 (2,2) |  |
| Orf19.5063 | Coi1 | Secreted protein, ciclopiroxolamine induced | 3 (2,3,2) | 1 (1) | Yes |
| Orf19.2706 | Crh11 | GPI-anchored cell wall transglycosylase | 3 (11,5,4) | 0 | Yes |
| Orf19.2531 | Csp37 | Hyphal cell wall protein | 2 (2,3) | 1 (1) |  |
| Orf19.6472 | Cyp1 | Peptidyl-prolyl cis-trans isomerase; cyclosporine A sensitive activity | 4 (9,9,9,6) | 3 (6,8,3) |  |
| Orf19.6402 | Cys3 | Cystathionine gamma-lyase; upregulated by alkaline, biofilm, amphotericin B, heavy metal (cadmium), stress, oxidative stress (via Cap1p) | 4 (3,2,3,2) | 2 (1,4) |  |
| Orf19.4777 | Dak2 | Putative dihydroxyacetone kinase; fluconazole-induced; caspofungin repressed | 3 (2,3,2) | 1 (2) |  |
| Orf19.4082 | Ddr48 | Immunogenic stress-associated protein; regulated by filamentous growth pathways; induced fenomyl, caspofungin, ketoconazole or azole-resistant strain | 4 (6,1,10,2) | 2 (5,4) |  |
| Orf19.7392 | Ded1 | Ortholog(s) have ATP-dependent RNA helicase activity | 3 (3,1,2) | 0 |  |
| Orf19.6702 | Ded81 | Putative tRNA-Asn synthetase | 2 (2,1) | 0 |  |
| Orf19.2407 | Dps1-1 | Putative tRNA-Asp synthetase | 2 (2,6) | 0 |  |
| Orf19.3374 | Ece1 | Cell elongation protein 1; Hyphal-specific protein; fluconazole-induced | 4 (14,15,6,10) | 3 (14,2,1) | Yes |
| Orf19.3010.1 | Ecm33 | GPI-anchored cell wall protein | 4 (6,4,3,2) | 2 (3,1) | Yes |
| Orf19.3838 | Efb1 | Translation elongation factor EF-1 beta; antigenic in mouse | 4 (5,7,6,4) | 3 (3,3,1) |  |
| Orf19.5788 | Eft2 | Elongation factor 2 (eEF2); GTPase; essential; highly expressed; target of sordarin antifungals; antigenic in human/mouse | 4 (16,17,16,5) | 3 (3,6,3) |  |
| Orf19.1154 | Egd1 | Putative GAL4 DNA-binding enhancer protein, biofilm induced | 4 (2,1,6,1) | 0 |  |
| Orf19.5858 | Egd2 | Putative nascent polypeptide associated complex protein alpha subunit | 4 (5,5,7,4) | 2 (2,1) |  |
| Orf19.7626 | Eif4E | Translation initiation factor eIF4E | 3 (1,1,3) | 0 |  |
| Orf19.731 | Emp46 | Protein similar to *S. cerevisiae* Emp46p | 3 (3,1,2) | 1 (1) | Yes |
| Orf19.3066 | Eng1 | Endo-1,3-beta-glucanase required for cell separation after division | 3 (4,7,1) | 1 (2) | Yes |
| Orf19.395 | Eno1 * | Enolase; binds host plasmin/plasminogen | 4 (27,31,31,12) | 3 (15,30,10) |  |
| Orf19.1591 | Erg10 | Acetyl-CoA acetyltransferse; role in ergosterol biosynthesis; changes in protein abundance associated with azole resistance; fluconazole or ketoconazole induced | 4 (3,3,4,3) | 0 |  |
| Orf19.7312 | Erg13 | 3-hydroxy-3-methylglutaryl coenzyme A synthase; ergosterol biosynthesis protein; amphotericin B, aspofungin repressed | 2 (3,3) | 0 |  |
| Orf19.7592 | Faa4 | Predicted acyl-Coa synthetase | 4 (4,3,4,1) | 0 |  |
| Orf19.979 | Fas1 | Beta subunit of fatty-acid synthase; fluconazole-induced; amphotericin B, caspofungin repressed; fungal-specific (no human or murine homolog) | 4 (5,1,1,3) | 0 |  |
| Orf19.5949 | Fas2 | Alpha subunit of fatty-acid synthase; fluconazole-induced; amphotericin B repressed | 3 (3,1,2) | 0 |  |
| Orf19.4618 | Fba1 * | Putative fructose-bisphosphate aldolase, enzyme of glycolysis; antigenic in murine/human infection; induced by fluconazole; binds human plasminogen | 4 (18,13,19,12) | 3 (11,11,4) |  |
| Orf19.7600 | Fdh3 | Glutathione-dependent formaldehyde dehydrogenase involved in glycine catabolism | 4 (1,3,2,1) | 0 |  |
| Orf19.2573 | Frs1 | Phenylalanyl-tRNA sinthetase; possible role in early cell wall biosynthesis | 2 (1,2) | 0 |  |
| Orf19.2960 | Frs2 | Putative tRNA-Phe synthetase | 2 (2,1) | 0 |  |
| Orf19.1153 | Gad1 | Putative glutamate decarboxylase; amphotericin B induced | 2 (9,1) | 1 (2) |  |
| Orf19.4899 | Gca1 | Possible adhesin; predicted extracellular or plasma membrane-associated glucoamylase | 3 (1,1,2) | 1 (1) | Yes |
| Orf19.7394 | Gda1 | Golgi membrane GDPase, required for wild-type O-mannosylation | 4 (6,6,3,3) | 2 (2,5) | Yes |
| Orf19.1618 | Gfa1 | Glucosamine-6-phosphate synthase | 4 (2,1,3,1) | 0 |  |
| Orf19.3182 | Gis2 | Putative transcription facto; null mutant exhibits sensitivity to sorbitol, 5-flurocytosine and cold temperatures | 4 (3,3,1,2) | 0 |  |
| Orf19.6285 | Glc7 | Putative catalytic subunit of type 1 serine/threonine protein phosphatase | 3 (2,1,1) | 0 |  |
| Orf19.6116 | Glk4 | Putative glucokinase | 3 (2,3,1) | 0 |  |
| Orf19.646 | Gln1 | Putative glutamate synthase | 3 (3,2,1) | 0 |  |
| Orf19.6257 | Glt1 | Putative glutamate synthase | 2 (2,1) | 0 |  |
| Orf19.251 | Glx3 | Glutathione-independent glyoxalase; binds human immunogloblulin E | 4 (5,3,15,3) | 2 (3,14) |  |
| Orf19.5024 | Gnd1 | Putative 6-phosphogluconate dehydrogenase; antigenic in mice | 4 (8,16,16,12) | 3 (10,8,5) |  |
| Orf19.691 | Gpd2 | Similar to glycerol 3-P dehydrogenase; binds to host complement regulator; binds human plasminogen | 2 (2,2) | 0 |  |
| Orf19.7021 | Gph1 * | Putative glycogen phosphorylase with a role in glycogen metabolism | 3 (1,7,2) | 0 |  |
| Orf19.4317 | Gre3 | Putative D-xylose reductase; antigenic in murine systemic infection | 2 (1,6) | 1 (3) |  |
| Orf19.4309 | Grp2 | Methylglyoxal reductase; regulation associated with azole resistance; induced in core stress response, fluphenazine, benomyl, or with long term fluconazole treatment; antigenic in humans | 3 (4,2,7) | 2 (4,1) |  |
| Orf19.437 | Grs1 | Putative tRNA-Gly synthetase | 4 (3,4,4,1) | 0 |  |
| Orf19.2929 | Gsc1 | Subunit of beta-1,3-glucan synthase | 2 (2,1) | 0 |  |
| Orf19.5493 | Gsp1 | Small RAN G-protein; essential | 4 (1,1,3,1) | 2 (1,1) |  |
| Orf19.2803 | Hem13 * | Coproporphyrinogen III oxidase; antigenic; possibly essential | 4 (6,3,4,2) | 1 (3) |  |
| Orf19.6327 | Het1 | Putative sphingolipid transfer protein involved in localization of glucosylceramide, which is important for virulence | 4 (1,2,1,2) | 2 (2,3) |  |
| Orf19.2020 | Hgt6 | Putative high-affinity major facilitator superfamily glucose transporter; core stress response, fluconazole-induced | 3 (2,3,2) | 0 |  |
| Orf19.1059 | Hhf1 | Putative histone H4; fluconazole induced; amphotericin B repressed | 2 (2,1) | 0 |  |
| Orf19.1061 | Hht21 | Putative histone H3; amphotericin B respressed | 2 (1,2) | 0 |  |
| Orf19.6645 | Hmo1 | Transcription factor that binds upstream of genes involved in hexose and ergosterol metabolism and in the cell cycle | 3 (1,1,4) | 0 |  |
| Orf19.2341 | Hnt1 | Protein with similarity to protein kinase C inhibitor-I | 4 (1,2,3,1) | 2 (1,2) |  |
| Orf19.2951 | Hom6 | Putative homoserine dehydrogenase | 4 (4,4,3,1) | 0 |  |
| Orf19.6387 | Hsp104 | Heat-shock protein; roles in biofilm and virulence; guanidine-insensitive; heat shock/stress induced; repressed in farnesol-treated biofilm | 3 (6,3,11) | 0 |  |
| Orf19.3160 | Hsp12 | Heat-shock protein; induced upon osmotic/oxidative/cadmium stress, fluphenazine treatment, low iron, CDR1 and CDR2 overexpression; repressed by Hog1p, flucytosine, elevated CO_2_ | 3 (5,4,7) | 3 (4,3,1) |  |
| Orf19.822 | Hsp21 | Unknown | 3 (1,7,1) | 1 (1) |  |
| Orf19.4980 | Hsp70 * | Binds beta-defensin; Hsp70 family protein chaperone; role in entry into host cells | 4 (22,22,24,19) | 3 (15,15,1) |  |
| Orf19.6515 | Hsp90 * | Essential chaperone, regulates several signal transduction pathways and temperature-induced morphogenesis; localizes to surface of hyphae | 4 (24,22,21,18) | 3 (13,11,2) |  |
| Orf19.6924 | Hta1 | Histone H2A; amphotericin B repressed; farnesol regulated | 2 (3,3) | 0 |  |
| Orf19.1051 | Hta2 | Putative histone H2A | 2 (3,3) | 1 (1) |  |
| Orf19.327 | Hta3 | Putative histone H2A; amphotericin B repressed; flucytosine induced | 2 (2,1) | 0 |  |
| Orf19.542 | Hxk2 | Hexokinase II; antigenic in human; fluconazole-induced | 4 (17,13,19,10) | 2 (8,12) |  |
| Orf19.4975 | Hyr1 | Hyphal-induced GPI-anchored cell wall protein | 4 (8,6,1,1) | 2 (1,9) | Yes |
| Orf19.2775 | Idi1 | Ortolog(s) have isopentenyl-diphosphate delta-isomerase activity | 4 (3,4,3,1) | 1 (2) |  |
| Orf19.5211 | Idp1 | Putative isocitrate dehydrogenase | 2 (1,2) | 0 |  |
| Orf19.3733 | Idp2 | Isocitrate dehydrogenase | 2 (1,4) | 0 |  |
| Orf19.5760 | Ihd1 | Putative GPI-anchored protein | 4 (3,3,2,2) | 3 (2,2,1) | Yes |
| Orf19.7585 | Ino1 * | Inositol-1-phosphate synthase; antigenic in human; repressed by caspofungin | 4 (13,12,16,17) | 3 (12,20,1) |  |
| Orf19.3590 | Ipp1 | Putative inorganic pyrophosphatase; antigenic; possibly an essential gene | 4 (9,10,11,12) | 2 (8,7) |  |
| Orf19.2792 | Ist2 | Ortolog(s) have lipid binding activity and cellular bud membrane, endoplasmic reticulum localization | 3 (2,2,1) | 0 |  |
| Orf19.2013 | Kar2 | Similar to chaperones of Hsp70p family; role in protein translocation into ER | 4 (10,14,15,6) | 2 (8,5) | Yes |
| Orf19.4755 | Kex2 | Subtilisin-like protease (proprotein convertase), processes aspartyl proteinase Sap2p; required for hyphal growth and wild-type virulence in mice | 4 (2,1,3,2) | 1 (2) | Yes |
| Orf19.7510 | Kin2 | Protein with similarity to *S. cerevisiae* Kin2p | 2 (1,2) | 0 |  |
| Orf19.6749 | Krs1 | Putative tRNA-Lys synthetase | 4 (5,2,7,2) | 0 |  |
| Orf19.6561 | Lat1 | Putative dihydrolipoamide acetyltransferse component (E2) of pyruvate dehydrogenase complex | 3 (1,3,1) | 2 (2,2) |  |
| Orf19.3358 | Lsc1 | Putative succinte-CoA ligase subunit; fluconazole-induced | 3 (2,1,1) | 0 |  |
| Orf19.3149 | Lsp1 | Eisosome component with a predicted role in endocytosis; caspofungin repressed; fungal-specific (no human or murine homolog) | 4 (8,4,12,4) | 1 (2) |  |
| Orf19.4506 | Lys22 | Putative homocitrate synthase; fungal-specific (no human or murine homolog) | 3 (2,2,3) | 0 |  |
| Orf19.3294 | Mbf1 | Putative transcriptional coactivator; caspofungin repressed | 3 (1,2,1) | 0 |  |
| Orf19.7239 | Mdg1 | Ortolog(s) have role in pheromone-dependent signal transduction involved in conjugation cellular fusion and membrane raft, plasma membrane localization | 4 (3,4,4,1) | 0 |  |
| Orf19.7481 | Mdh1 | Malate dehidrogenase, mitochondrial; antigenic during murine and human infection | 4 (2,1,12,1) | 0 | Yes |
| Orf19.4602 | Mdh1-1 | Malate dehydrogenase | 4 (4,1,5,2) | 0 |  |
| Orf19.5645 | Met15 | O-acetylhomoserine O-acetylserine sulfhydrylase in sulphur amino acid biosynthesis; immunogenic | 4 (5,4,8,2) | 2 (2,3) |  |
| Orf19.2551 | Met6 | Essential 5-methyltetrahydropteroyltriglutamate-homocysteine methyltransferase; antigenic during murine or human systemic infection | 4 (14,19,29,17) | 3 (13,11,2) |  |
| Orf19.2364 | Mis11 | Similar to precursor of mitochondrial C1-tetrahydrofolate synthase; fluconazole-induced; putative protein of glycine catabolism | 2 (1,3) | 1 (1) |  |
| Orf19.2416.1 | Mlc1 | Protein with microtubule-dependent localization to the Spitzendhorper, also localizes to cytokinetic ring in hyphae | 4 (4,3,2,2) | 2 (3,2) |  |
| Orf19.6692 | Mnn26 | Putative mannosyltransferase (PMT) | 2 (3,3) | 0 | Yes |
| Orf19.1665 | Mnt1 | Alpha-1,2-mannosyl transferase | 2 (2,3) | 0 | Yes |
| Orf19.1779 | Mp65 | Cell surface mannoprotein | 4 (6,9,10,6) | 3 (5,6,6) | Yes |
| Orf19.1490 | Msb2 | Mucin family adhesion-like protein; cell wall damage sensor | 4 (7,4,3,2) | 3 (2,2,2) | Yes |
| Orf19.2435 | Msi3 | Essential antigenic HSP70 family protein, required for fluconazole resistance and calcineurin-dependent transcription | 4 (4,3,13,1) | 2 (1,3) |  |
| Orf19.6105 | Mvd | Mevalonate diphosphate decarboxylase; possible drug target; transcriptionally regulated by yeast-hyphal switch, growth phase, antifungals | 2 (2,1) | 0 |  |
| Orf19.2028 | Mxr1 | Putative methionine sulfoxide reductase; flucytosine induced | 4 (2,1,2,3) | 1 (1) |  |
| Orf19.5015 | Myo2 | Class V myosin | 2 (3,5) | 0 |  |
| Orf19.4623.3 | Nhp6A | Putative non-histone chromatin component; amphotericin B, caspofungin repressed | 3 (2,2,3) | 0 |  |
| Orf19.3138 | Nop1 | Nucleolar protein; flucytosine induced | 3 (1,2,3) | 0 |  |
| Orf19.6570 | Nup | Nucleoside permease | 3 (2,2,1) | 1 (3) | Yes |
| Orf19.1052 | Orf19.1052 | Predicted histone H2B | 4 (2,1,2,2) | 1 (1) |  |
| Orf19.1086 | Orf19.1086 | Ortholog(s) have role in protein deubiquitination, regulation of ER to Golgi vesicle-mediated transport and Golgi to ER, ribophagy | 2 (1,2) | 0 |  |
| Orf19.1564 | Orf19.1564 | Plasma membrane-localized protein of unknown function | 2 (1,2) | 0 | Yes |
| Orf19.1862 | Orf19.1862 | Possible estress protein | 4 (4,4,4,3) | 2 (1,1) |  |
| Orf19.1946 | Orf19.1946 | Similar to an aldose 1-epimerase-related protein; antigenic during murine systemic infection | 2 (1,3) | 1 (2) |  |
| Orf19.200 | Orf19.200 | Ortolog(s) have RNA binding | 3 (1,3,1) | 1 (1) |  |
| Orf19.2125 | Orf19.2125 | Protein of unknown function | 1 (2) | 2 (2,1) |  |
| Orf19.2269 | Orf19.2269 | Putative 3-phosphaserine phosphatase; fungal-specific (no human or murine homolog) | 2 (1,2) | 2 (1,1) |  |
| Orf19.2286 | Orf19.2286 | Putative deoxyhypusine hydroxylase; required for biofilm formation; ketoconazole-induced | 3 (2,2,3) | 0 |  |
| Orf19.2296 | Orf19.2296 | Predicted mucin-like protein; ketoconazole-induced; fluconazole-downregulated | 2 (3,11) | 0 |  |
| Orf19.2478.1 | Orf19.2478.1 | Protein L7 60S large ribosomal subunit | 4 (6,3,5,4) | 2 (2,1) |  |
| Orf19.2769 | Orf19.2769 | Putative protease B inhibitor; hyphal-induced expression | 4 (1,1,4,2) | 2 (2,2) | Yes |
| Orf19.3003 | Orf19.3003 | Putative lipid-binding protein with a predicted role in calcium-dependent phospholipids-binding | 3 (1,1,2) | 1 (1) |  |
| Orf19.3037 | Orf19.3037 | Putative poly(A)-binding protein | 3 (1,1,5) | 0 |  |
| Orf19.3053 | Orf19.3053 | Biofilm-induced gene; identified in extracts from biofilm and planktonic cells | 3 (1,1,4) | 1 (4) |  |
| Orf19.3061.1 | Orf19.3061.1 | Orotolog(s) of *S. cerevisiae* Rsp22Ap and Rps22Bp | 3 (3,3,3) | 1 (2) |  |
| Orf19.3247 | Orf19.3247 | Putative ortholog of *S. Cerevisiae* Laa1p | 2 (1,2) | 0 |  |
| Orf19.3354 | Orf19.3354 | Otholog(s) have structural constituent of ribosome activity and 90S preribosome, cytosolic small ribosomal subunit, nucleolus localization | 4 (9,9,11,4) | 2 (3,4) |  |
| Orf19.3475 | Orf19.3475 | Described as a Gag-related protein; downregulation correlates with clinical development of fluconazole resistance | 4 (3,4,5,3) | 2 (2,1) |  |
| Orf19.3499 | Orf19.3499 | Secreted protein; fluconazole-induced | 2 (3,6) | 0 | Yes |
| Orf19.3572.3 | Orf19.3572.3 | Ribosomal 60S subunit protein L31B | 4 (3,3,4,3) | 3 (2,2,1) |  |
| Orf19.3681 | Orf19.3681 | Ortholog(s) have Ran guanyl-nucleotide exchange factor activity, protein transporter activity | 3 (1,2,1) | 1 (1) |  |
| Orf19.3690.2 | Orf19.3690.2 | Ribosomal 60S subunit protein; Spider biofilm repressed | 3 (4,5,2) | 0 |  |
| Orf19.3932 | Orf19.3932 | Predicted RNA binding protein; stationary phase enriched; induced in core caspofungin response; induced by ketoconazole and by hypoxia | 3 (1,4,3) | 2 (3,2) |  |
| Orf19.4149.1 | Orf19.4149.1 | Protein component of the small (40S) ribosomal subunit; Spider biofilm repressed | 4 (5,2,2,2) | 2 (1,1) |  |
| Orf19.4216 | Orf19.4216 | Putative heat shock protein; transcription is increased in populations of cells exposed to fluconazole over multiple generations | 4 (5,4,7,4) | 0 |  |
| Orf19.4246 | Orf19.4246 | Protein with similarity to *S. cerevisiae* Ykr070wp; induced during cell wall regeneration; possibly essential | 2 (2,4) | 1 (1) |  |
| Orf19.4796 | Orf19.4796 | Putative eIF-4E-binding repressor of CAP-dependent translation | 2 (1,1,2) | 0 |  |
| Orf19.5281 | Orf19.5281 | Predicted essential RNA-binding G protein | 4 (5,2,6,1) | 0 |  |
| Orf19.5620 | Orf19.5620 | Stationary phase enriched protein; Gcn4-regulated; flow model biofilm induced | 2 (2,1) | 0 |  |
| Orf19.5682 | Orf19.5682 | Ortholog(s) have protein binding, bridging, protein transporter activity | 2 (1,2) | 0 |  |
| Orf19.5773 | Orf19.5773 | Putative dipeptidyl-peptidase III | 4 (2,4,4,1) | 2 (1,1) |  |
| Orf19.5943.1 | Orf19.5943.1 | Putative protein of unknown function | 3 (6,3,4) | 1 (1) |  |
| Orf19.6160 | Orf19.6160 | Ortholog(s) have role in eisosome assembly and esisosome, membrane raft, plasma membrane localization | 3 (1,2,5,) | 2 (1,1) |  |
| Orf19.6403 | Orf19.6403 | Ortholog(s) have adenyl-nucleotide exchange factor activity, role in SRP-dependent cotranslational protein targeting to membrane | 3 (1,2,4) | 0 | Yes |
| Orf19.6415.1 | Orf19.6415.1 | Ortholog(s) have structural constituent of ribosome activity and cytosolic small ribosomal subunit, nucleus localization | 4 (2,2,1,1) | 1 (1) |  |
| Orf19.6507 | Orf19.6507 | Stationary phase enriched protein | 3 (1,1,2) | 0 |  |
| Orf19.6553 | Orf19.6553 | Membrane-localized protein; possibly secreted; fluconazole-induced | 3 (3,4,1) | 2 (1,2) | Yes |
| Orf19.6701 | Orf19.6701 | Protein with similarity to amino acid-tRNA ligase | 4 (2,3,4,1) | 1 (2) |  |
| Orf19.6882.1 | Orf19.6882.1 | Ribosomal 60S subunit protein; Spider biofilm repressed | 4 (2,2,2,2) | 2 (1,1) |  |
| Orf19.7085 | Orf19.7085 | Induced in core stress response | 3 (2,1,3) | 0 |  |
| Orf19.7297 | Orf19.7297 | Putative cystathionine gamma-synthase | 2 (1,2) | 0 |  |
| Orf19.7368 | Orf19.7368 | Ortolog(s) have mRNA binding, poly(U) RNA binding activity and role in nuclear-transcribed mRNA catabolic process, nonsense-mediated decay, regultation of mRNA stability, stress granule assembly | 2 (2,1) | 1 (1) |  |
| Orf19.8 | Orf19.8 | Ortholog of *C. dubliniensis CD36* | 0 | 2 (2,1) | Yes |
| Orf19.86 | Orf19.86 | Putative glutathione peroxidase | 2 (1,2) | 0 |  |
| Orf19.7514 | Pck1 | Phosphoenolpyruvate carboxykinase; induced by fluconazole, phagocytosis, H_2_O_2_, biofilm formation | 3 (4,3,9) | 0 |  |
| Orf19.3097 | Pda1 | Putative pyruvate dehydrogenase alpha chain; fluconazole-induced | 2 (2,1) | 0 |  |
| Orf19.2877 | Pdc11 * | Pyruvate decarboxylase; antigenic; on hyphal not yeast cell surface | 4 (19,20,23,22) | 3 (19,17,11) |  |
| Orf19.5130 | Pdi1 | Putative protein disulfide-isomerase; antigenic in human infection | 4 (10,11,15,4) | 2 (4,1) | Yes |
| Orf19.930 | Pet9 | Mitochondrial ADP/ATP carrier protein involved in ATP biosynthesis; possible lipid raft component; flucytosine induced; ketoconazole-induced | 4 (4,3,2,2) | 1 (1) | Yes |
| Orf19.3967 | Pfk1 | Phosphofructokinase alpha subunit, Pfk1p-Pfk2p heteromultimer; activated by fructose 2,6-biphosphate, AMP, inhibited by ATP; fluconazole-induced | 3 (4,1,10) | 0 |  |
| Orf19.6540 | Pfk2 | Phosphofructokinase beta subunit, a Pfk1p-Pfk2p heteromultimer; fructose 2,6-bisphosphate, AMP activated; ATP inhibited; phagocytosis, hyphal repressed; fluconazole-induced; | 3 (4,3,6) | 0 |  |
| Orf19.5076 | Pfy1 | Profilin, functional homolog of *S. cerevisiae* Pfy1p | 4 (4,3,4,5) | 2 (4,4) |  |
| Orf19.5674 | Pga10 | GPI anchored membrane protein; utilization of hemin and hemoglobin for Fe in host | 3 (2,2,2) | 0 | Yes |
| Orf19.4035 | Pga4 | GPI-anchored cell surface protein | 4 (8,4,9,3) | 1 (1) | Yes |
| Orf19.2451 | Pga45 | Putative GPI-anchored cell wall protein | 4 (5,6,2,2) | 2 (3,1) | Yes |
| Orf19.6217 | Pga63 | Component COPII vesicle coat; required for vesicle formation in ER to Golgi transport | 4 (2,1,5,2) | 0 |  |
| Orf19.3888 | Pgi1 | Putative glucose-6-phosphate isomerase, enzyme of glycolisis; antigenic in human | 4 (8,8,20,10) | 2 (11,8) |  |
| Orf19.3651 | Pgk1 * | Phosphoglycerate kinase; binds human plasminogen | 4 (17,19,28,21) | 3 (20,21,9) |  |
| Orf19.2841 | Pgm2 | Ortholog of *S. cerevisiae* Pgm2; flow model biofilm induced | 4 (4,5,5,2) | 0 |  |
| Orf19.3829 | Phr1 | Cell surface glycosidase; role in systemic, not vaginal virulence (neutral, not low pH); high pH or filamentation induced | 4 (21,26,18,16) | 3 (10,13,1) | Yes |
| Orf19.6081 | Phr2 | Glycosidase; role in vaginal not systemic infection (low pH not neutral); low pH, high iron, fluconazole, Hap43-induced | 3 (3,3,3) | 0 | Yes |
| Orf19.778 | Pil1 | Eisosome component with a predicted role in endocytosis; echinocandin-binding protein; fungal specific (no human or murine homolog) | 4 (7,3,10,4) | 3 (4,2,1) |  |
| Orf19.220 | Pir1 | 1,3-beta-glucan-linked structural cell wall protein | 4 (2,2,2,1) | 3 (1,2,1) | Yes |
| Orf19.6594 | Plb3 | GPI-anchored cell surface phospholipase B | 3 (3,3,3) | 1 (1) | Yes |
| Orf19.1442 | Plb4.5 | Phospholipase B | 2 (6,5) | 0 | Yes |
| Orf19.5383 | Pma1 | Plasma membrane H(+)-ATPase; highly expressed, comprising 20-40% of total plasma membrane protein; fluconazole induced; caspofungin repressed | 4 (9,9,11,5) | 2 (2,2) |  |
| Orf19.1390 | Pmi1 | Phosphomannose isomerase | 2 (2,1) | 1 (4) |  |
| Orf19.2937 | Pmm1 | Phosphomannomutase; enzyme of O- and N-linked mannosylation | 4 (5,3,5,5) | 2 (3,3) |  |
| Orf19.6812 | Pmt2 | Protein mannosyltransferase (PMT) with roles in hyphal growth and drug sensitivity | 2 (2,1) | 0 |  |
| Orf19.5793 | Pr26 | Protein with similarity to proteasomal 26S regulatory subunit of *S. cerevisiae, H. sapiens* | 2 (1,3) | 0 |  |
| Orf19.3111 | Pra1 | Cell surface protein that sequesters zinc from host tissue; enriched at hyphal tips; binds to host complement regulator; immunogenic in mouse; binds human plasminogen | 2 (3,1) | 2 (2,2) | Yes |
| Orf19.5180 | Prx1 | Thioredoxin peroxidase; present in exponential and stationary growth phase yeast cultures | 4 (6,3,6,2) | 2 (2,1) |  |
| Orf19.2241 | Pst1 | Putative 1,4-benzoquinone reductase; hyphal-induced | 2 (2,1) | 1 (1) | Yes |
| Orf19.5285 | Pst3 | Putative flavodoxin; fungal-specific (no human or murine homolog) | 3 (3,4,3) | 2 (1,1) |  |
| Orf19.1760 | Ras1 | RAS signal transduction GTPase; regulates cAMP and MAP kinase pathways; role in hyphal induction, virulence, apoptosis; plasma membrane localized | 3 (2,3,2) | 0 |  |
| Orf19.6452 | Rbp1 | Peptidyl-prolyl cis-trans isomerase; rapacycin-binding protein | 3 (1,1,3) | 3 (1,1,1) |  |
| Orf19.1327 | Rbt1 | Cell wall protein with similarity to Hwp1; required for virulence | 4 (9,8,5,14) | 3 (11,10,12) | Yes |
| Orf19.5636 | Rbt5 | GPI-linked cell wall protein | 4 (2,2,3,2) | 3 (2,1,1) | Yes |
| Orf19.7350 | Rct1 | Fluconazole-induced protein | 4 (3,5,6,5) | 1 (5) |  |
| Orf19.5968 | Rdi1 | Putative rho GDP dissociation inhibitor | 2 (1,2) | 0 |  |
| Orf19.4236 | Ret2 | Delta subunit of the coatomer complex (COPI); coats Golgi-derived transport vesicles | 2 (2,2) | 0 |  |
| Orf19.5305 | Rhd3 | GPI-anchored yeast-associated cell wall protein | 2 (3,2) | 0 | Yes |
| Orf19.2843 | Rho1 | Small GTPase of Rho family; regulates beta-1,3-glucan synthesis activity and binds Gsc1p | 4 (7,5,4,1) | 1 (1) |  |
| Orf19.5437 | Rhr2 | Glycerol 3-phosphatase; roles in osmotic tolerance, glycerol accumulation in response to salt; required for biofilm formation; biofilm-induced | 2 (3,2) | 0 |  |
| Orf19.5801 | Rnr21 | Ribonucleoside-diphosphate reductse; fluconazole or flucystosine induced | 2 (3,1) | 0 |  |
| Orf19.2935 | Rpl10 | Ribosomal protein L10 | 4 (6,5,7,4) | 3 (1,4,2) |  |
| Orf19.3465 | Rpl10A | Predicted ribosomal protein | 2 (1,2) | 0 |  |
| Orf19.2232 | Rpl11 | Ribosomal protein; repressed by phagocytosis | 4 (4,3,5,2) | 1 (1) |  |
| Orf19.1635 | Rpl12 | Ribosomal protein L12 | 4 (3,5,7,1) | 2 (1,2) |  |
| Orf19.2994 | Rpl13 | Putative ribosomal subunit; antigenic during murine systemic infection | 4 (2,1,2,2) | 0 |  |
| Orf19.4931.1 | Rpl14 | Ribosomal protein L14 | 4 (5,3,11,3) | 1 (1) |  |
| Orf19.493 | Rpl15A | Putative ribosomal protein | 4 (3,3,4,3) | 2 (1,3) |  |
| Orf19.4490 | Rpl17B | Ribosomal protein L17 | 4 (4,3,5,2) | 2 (2,2) |  |
| Orf19.5982 | Rpl18 | Predicted ribosomal protein | 4 (3,1,4,1) | 2 (2,2) |  |
| Orf19.5904 | Rpl19A | Ribosomal protein L19 | 4 (2,3,4,2) | 1 (3) |  |
| Orf19.2309.2 | Rpl2 | Putative 60S ribosomal protein L2 | 4 (6,6,6,2) | 2 (2,1) |  |
| Orf19.4632 | Rpl20B | Ribosomal protein L20 | 4 (2,4,7,3) | 1 (1) |  |
| Orf19.840 | Rpl21A | Putative ribosomal protein | 3 (3,3,5) | 1 (2) |  |
| Orf19.3504 | Rpl23A | Ribosomal protein | 4 (3,2,5,2) | 2 (2,1) |  |
| Orf19.3789 | Rpl24A | Predicted ribosomal protein | 3 (2,4,2) | 3 (2,2,2) |  |
| Orf19.687.1 | Rpl25 | Putative rRNA-binding ribosomal protein component of the 60S ribosomal subunit | 4 (2,2,1,2) | 0 |  |
| Orf19.5225.2 | Rpl27A | Ribosomal protein L27 | 4 (4,2,3,4) | 3 (1,2,2) |  |
| Orf19.2864.1 | Rpl28 | Putative ribosomal protein | 4 (5,3,2,3) | 1 (1) |  |
| Orf19.1601 | Rpl3 | Putative ribosomal protein, large subunit | 4 (6,4,9,5) | 3 (5,6,3) |  |
| Orf19.3788.1 | Rpl30 | Similar to *S. cerevisiae* L30 ribosomal subunit | 4 (4,2,4,2) | 2 (1,1) |  |
| Orf19.3415.1 | Rpl32 | Component of the large (60S) ribosomal subunit | 4 (2,1,2,2) | 0 |  |
| Orf19.5964.2 | Rpl35 | Ribosomal protein | 4 (1,2,6,2) | 1 (1) |  |
| Orf19.2111.2 | Rpl38 | 60S ribosomal protein subunit | 3 (1,3,3) | 2 (1,1) |  |
| Orf19.827.1 | Rpl39 | Ribosomal protein L39 | 3 (1,1,2) | 1 (1) |  |
| Orf19.7217 | Rpl4B | Ribosomal protein 4B | 4 (6,7,11,6) | 3 (3,1,3) |  |
| Orf19.6541 | Rpl5 | Ribosomal protein | 4 (5,5,9,5) | 1 (1) |  |
| Orf19.3003.1 | Rpl6 | Protein similar to *S. cerevisiae* ribosomal subunit, Rpl6p | 4 (8,7,8,3) | 1 (2) |  |
| Orf19.2311 | Rpl82 | Predicted ribosomal protein | 1 (7) | 3 (3,2,2) |  |
| Orf19.6002 | Rpl8B | Predicted ribosomal protein; transcription is regulated upon yeast-hyphal switch | 4 (12,8,10,4) | 0 |  |
| Orf19.236 | Rpl9B | Ribosomal protein L9 | 4 (2,2,4,1) | 0 |  |
| Orf19.7015 | Rpp0 | Putative ribosomal protein; antigenic in mouse | 4 (3,4,6,2) | 2 (1,2) |  |
| Orf19.6403.1 | Rpp2A | Conserved acidic ribosomal protein, likely involved in regulation of translation elongation; interacts with Rpp1Bp | 3 (1,1,2) | 0 | Yes |
| Orf19.3002 | Rps1 | Putative ribosomal protein 10 of the 40S subunit, elicits a host antibody response during infection | 3 (5,7,10) | 2 (2,4) |  |
| Orf19.2179.2 | Rps10 | Ribosomal protein S10; downregulated in the presence of human whole blood or polymorphonuclear (PMC) cells | 4 (1,2,1,1) | 1 (1) |  |
| Orf19.6785 | Rps12 | Acidic ribosomal protein S12 | 3 (2,3,3) | 3 (3,1,1) |  |
| Orf19.4193.1 | Rps13 | Putative ribosomal protein of the small subunit | 4 (3,2,4,2) | 2 (2,2) |  |
| Orf19.6265.1 | Rps14B | Putative ribosomal protein | 4 (2,1,6,2) | 1 (2) |  |
| Orf19.5927 | Rps15 | Putative ribosomal protein | 4 (3,3,3,3) | 2 (2,3) |  |
| Orf19.2994.1 | Rps16A | Putative 40S ribosomal subunit | 3 (4,2,8) | 1 (1) |  |
| Orf19.2329.1 | Rps17B | Ribosomal protein 17B | 3 (1,2,2) | 2 (1,1) |  |
| Orf19.7018 | Rps18 | Predicted ribosomal protein | 4 (4,2,6,2) | 1 (2) |  |
| Orf19.5996.1 | Rps19A | Putative ribosomal protein S19 | 4 (5,5,5,3) | 3 (2,1,1) |  |
| Orf19.6375 | Rps20 | Putative ribosomal protein | 3 (6,5,6) | 1 (1) |  |
| Orf19.3334 | Rps21 | Predicted ribosomal protein | 4 (6,5,10,2) | 2 (1,1) |  |
| Orf19.3325.3 | Rps21B | Protein similar to ribosomal protein S21 | 4 (1,1,2,1) | 2 (3,1) |  |
| Orf19.6253 | Rps23A | Putative ribosomal protein | 3 (1,1,2) | 1 (1) |  |
| Orf19.5466 | Rps24 | Predicted ribosomal protein | 4 (4,3,4,3) | 2 (3,1) |  |
| Orf19.6663 | Rps25B | Ribosomal protein | 4 (3,2,3,3) | 0 |  |
| Orf19.1470 | Rps26A | Predicted ribosomal protein | 3 (2,1,2) | 0 |  |
| Orf19.6286.2 | Rps27 | Putative ribosomal protein | 4 (3,1,4,2) | 2 (1,1) |  |
| Orf19.7048.1 | Rps28B | Putative ribosomal protein S28B | 4 (2,1,3,3) | 0 |  |
| Orf19.6312 | Rps3 | Ribosomal protein S3 | 4 (10,8,9,3) | 2 (2,4) |  |
| Orf19.4336 | Rps5 | Ribosomal protein S5 | 4 (6,4,5,6) | 3 (2,3,1) |  |
| Orf19.4660 | Rps6A * | Ribosomal protein 6A | 4 (3,2,6,3) | 1 (1) |  |
| Orf19.1700 | Rps7A | Ribosomal protein S7 | 4 (7,6,8,4) | 3 (2,1,1) |  |
| Orf19.6873 | Rps8A | Small 40S ribosomal subunit protein | 4 (4,5,7,6) | 2 (2,2) |  |
| Orf19.838.1 | Rps9B | Predicted ribosomal protein | 4 (2,3,5,3) | 1 (1) |  |
| Orf19.7124 | Rvs161 | Protein required for endocytosis; contains a BAR domain, which is found in protein sinvolved in membrane curvature | 2 (2,1) | 0 |  |
| Orf19.3911 | Sah1 | Putative S-adenosyl-L-homocysteine hydrolase; antigenic in human | 4 (6,7,15,8) | 3 (6,3,2) |  |
| Orf19.657 | Sam2 * | S-adenosylmethionine synthetase; localizes to surface of hyphal cells | 4 (6,4,8,7) | 2 (4,3) |  |
| Orf19.5585 | Sap5 | Secreted aspartyl proteinase; assessment of virulence role complicated by URA3 effects; expressed during infection | 2 (3,2) | 0 | Yes |
| Orf19.6928 | Sap9 | Secreted aspartyl protease | 2 (3,4) | 0 | Yes |
| Orf19.3462 | Sar1 | Functional homolog of *S. cerevisiae* Sar1p, which is required for ER-to-Golgi protein transport | 3 (2,2,3) | 1 (1) |  |
| Orf19.5854 | Sbp1 | Similar to RNA binding proteins | 4 (3,2,2,1) | 2 (1,2) |  |
| Orf19.4928 | Sec2 | Guanyl-nucleotide exchange factor for the small G-protein Sec4p | 2 (2,1) | 0 |  |
| Orf19.4732 | Sec24 | Protein with a possible role in ER to Golgi transport | 2 (1,2) | 0 |  |
| Orf19.528 | Sec26 | Secretory vesicles coatomer complex protein | 2 (1,4) | 0 |  |
| Orf19.2571 | Sec4 | Small GTPase of Rab family; role in post-Golgi secretion | 4 (7,4,1,3) | 0 |  |
| Orf19.5484 | Ser1 | Putative 3-phosphoserine aminotransferase | 3 (2,1,2) | 1 (1) |  |
| Orf19.5263 | Ser33 | Predicted enzyme of amino acid biosynthesis | 2 (2,5) | 0 |  |
| Orf19.269 | Ses1 | Seryl-tRNA synthetase | 4 (4,2,3,1) | 0 |  |
| Orf19.4089 | Sgt1 | Putative co-chaperone protein with a predicted role in kinetochore assembly | 3 (2,1,2) | 0 |  |
| Orf19.5823 | Sgt2 | Putative small tetratricopeptide repeat (TPR)-containing protein | 4 (1,1,2,1) | 0 |  |
| Orf19.5750 | Shm2 | Cytoplasmic serine hydroxymethyltransferase; antigenic in human | 3 (6,2,3) | 1 (1) |  |
| Orf19.7569 | Sik1 | Putative U3 snoRNP protein | 3 (1,1,3) | 0 |  |
| Orf19.5032 | Sim1 | Putative adhesion-like protein involved in cell wall maintenance; secreted beta-glucosidase | 3 (3,2,1) | 1 (1) | Yes |
| Orf19.4427 | Skp1 | Putative subunit D of kinetochore protein complex CBF3 | 4 (1,2,4,1) | 1 (1) |  |
| Orf19.6763 | Slk19 | Alkaline-induced protein of plasma membrane | 3 (2,1,3) | 0 |  |
| Orf19.670 | Smt3 | SUMO, small ubiquitin-like protein | 4 (1,2,1,2) | 2 (1,1) |  |
| Orf19.2947 | Snz1 | Stationary phase protein | 4 (6,7,12,3) | 2 (3,3) |  |
| Orf19.2770.1 | Sod1 | Cytosolic copper- and zinc-containing superoxide dismutase, involved in protection from oxidative stress and required for full virulence | 4 (3,2,3,4) | 3 (4,2,1) |  |
| Orf19.2060 | Sod5 | Copper- and zinc-containing superoxide dismutase | 4 (4,3,5,4) | 2 (4,2) | Yes |
| Orf19.6190 | Srb1 * | Essential GDP-mannose pyrophosphorylase | 4 (5,6,7,4) | 2 (2,1) | Yes |
| Orf19.1065 | Ssa2 * | Binds histatin 5 and beta-defensin; Hsp70 family protein chaperone | 4 (25,23,23,17) | 3 (12,14,1) |  |
| Orf19.6367 | Ssb1 * | HSP70 family heat shock protein | 4 (15,16,21,14) | 3 (8,12,1) |  |
| Orf19.1896 | Ssc1 * | 70-kDa heat shock protein | 4 (4,3,2,2) | 0 |  |
| Orf19.1376 | Sso2 | Plasma membrane t-SNARE; involved in fusion of secretory vesicles at the plasma membrane | 3 (1,2,2) | 0 |  |
| Orf19.7030 | Ssr1 | Beta-glucan associated ser/thr rich cell-wall protein | 4 (2,2,2,1) | 3 (1,1,1) | Yes |
| Orf19.3812 | Ssz1 * | Putative HSP70 chaperone | 3 (1,4,4) | 0 |  |
| Orf19.3192 | Sti1 | Possibly involved Cdc37p chaperone activity; biofilm induced | 4 (7,7,2,3) | 0 |  |
| Orf19.5647 | Sub2 | Putative TREX complex component with a predicted role in nuclear mRNA export | 3 (2,1,2) | 1 (2) |  |
| Orf19.6213 | Sui2 | Translation initation factor eIF2, alpha chain | 2 (1,4) | 0 |  |
| Orf19.3414 | Sur7 | Protein required for normal cell wall, plasma membrane | 4 (2,3,1,2) | 1 (1) | Yes |
| Orf19.4371 | Tal1 | Transaldolase; biofilm induced | 4 (16,12,20,17) | 3 (10,13,1) |  |
| Orf19.6814 | Tdh3 * | NAD-linked glyceraldehyde-3-phosphate dehydrogenase; binds fibronectin, laminin and plasminogen | 4 (22,22,28,17) | 3 (13,13,11) |  |
| Orf19.1435 | Tef1 * | Translation elongation factor 1-alpha, detected at the cell surface; binds human plasminogen | 4 (15,13,17,13) | 2 (10,13) |  |
| Orf19.4233 | Thr4 | Putative threonine synthase | 3 (3,3,2) | 0 |  |
| Orf19.3324 | Tif | Translation initiation factor; antigenic in human | 4 (8,5,10,5) | 2 (3,4) |  |
| Orf19.3423 | Tif3 | Putative translation initiation factor | 2 (1,3) | 1 (1) |  |
| Orf19.3599 | Tif4631 | Putative translation initiation factor eIF4G | 3 (2,1,2) | 0 |  |
| Orf19.5112 | Tkl1 * | Putative transketolase | 4 (7,7,12,7) | 3 (5,6,1) |  |
| Orf19.3268 | Tma19 | Cell wall protein | 4 (2,2,2,1) | 0 |  |
| Orf19.3700 | Tom70 | Ortholog(s) have mitochondrion targeting sequence binding, protein channel activity and role in protein import into mitochondrial inner membrane | 3 (2,2,4) | 0 | Yes |
| Orf19.1690 | Tos1 | Protein similar to alpha agglutinin anchor subunit; secreted | 4 (7,6,4,4) | 3 (3,6,2) | Yes |
| Orf19.6745 | Tpi1 * | Triose-phosphate isomerase; antigenic in mouse or human | 4 (10,5,9,2) | 3 (5,7,3) |  |
| Orf19.6414.3 | Tpm2 | Putative tropomyosin isoform 2 | 4 (7,8,10,5) | 2 (6,4) |  |
| Orf19.4290 | Trr1 | Thioredoxin reductase | 3 (1,3,1) | 1 (1) |  |
| Orf19.7611 | Trx1 | Thioredoxin, involved in response to reactive oxygen species; amphtericin B, caspofungin repressed | 4 (1,1,2,1) | 2 (2,3) |  |
| Orf19.7417 | Tsa1 * | TSA/alkyl hydroperoxide peroxidase C (AhPC) family protein; binds human plasminogen | 4 (6,4,9,10) | 3 (5,4,2) |  |
| Orf19.6059 | Ttr1 | Putative glutaredoxin; described as a glutathione reductase; required for virulence in mouse model | 2 (2,1) | 0 |  |
| Orf19.7308 | Tub1 | Alpha-tubulin; farnesol-upregulated in biofilm | 3 (5,3,7) | 0 |  |
| Orf19.6034 | Tub2 | Beta-tubulin; fluconazole-induced | 3 (3,2,5) | 2 (2,1) |  |
| Orf19.6109 | Tup1 | Regulates phase switching, roles in germ tube induction, farnesol response | 3 (1,1,3) | 0 |  |
| Orf19.7438 | Uba1 | Ubiquitin-activating enzyme | 2 (2,5) | 0 |  |
| Orf19.3087 | Ubi3 | Fusion of ubiquitin with ribosomal S34 protein of the small ribosomal subunit | 2 (2,2) | 0 | Yes |
| Orf19.1738 | Ugp1 * | Possible UTP-glucose-1-phosphaturidyl transferase | 3 (5,5,7) | 0 |  |
| Orf19.2360 | Ura2 | Putative bifunctional carbamoylphosphate synthetase-aspartate transcarbamylase; flucytosine induced | 3 (1,1,2) | 0 |  |
| Orf19.1671 | Utr2 | Putative GPI anchored cell wall glycosidase | 2 (2,2) | 1 (1) | Yes |
| Orf19.1295 | Vas1 | Putative tRNA-Val synthetase | 3 (3,1,2) | 0 |  |
| Orf19.2598 | Vma4 | H+ transporting ATPase E chain; caspofungin repressed | 2 (1,2) | 0 |  |
| Orf19.1949 | Vps1 | Dynamin-family GTPase-related protein | 3 (9,8,3) | 0 |  |
| Orf19.3548.1 | Wh11 | Cytoplasmic protein expressed specifically in white phase yeast cells, expression in opaque cells increases virulence | 3 (3,2,6) | 1 (1) |  |
| Orf19.7676 | Xyl2 | D-xylulose reductase; immunogenic in mice; soluble protein in hyphae | 2 (1,2) | 0 |  |
| Orf19.754 | Ybn5 | Ortholog(s) have ATPase activity and cytosol, nucleus localization | 3 (4,2,6) | 0 |  |
| Orf19.506 | Ydj1 | Putative type I HSP40 co-chaperone; heavy metal (cadmium) stress-induced | 3 (2,2,1) | 0 |  |
| Orf19.2974 | Ykt6 | Putative protein of the vacuolar SNARE complex | 3 (2,1,2) | 0 |  |
| Orf19.4311 | Ynk1 | Nucleoside diphosphate kinase (NDP kinase); soluble protein in hyphae; flucytosine induced | 3 (3,3,6) | 3 (4,4,1) |  |
| Orf19.6481 | Yps7 | Putative aspartic-type endopeptidase | 3 (2,1,1) | 2 (2,1) | Yes |
| Orf19.3052 | Ypt1 | Functional homolog of *S. cerevisiae* Ypt1p, which is an essential small Ras-type GTPase involved in protein secretion at ER-toGolgi | 3 (3,2,2) | 2 (1,1) |  |
| Orf19.2622 | Ypt31 | Protein required for resistance to toxic ergosterol analog | 3 (3,5,1) | 0 |  |
| Orf19.7477 | Yrb1 | Functional homolog of *S. cerevisiae* Yrb1p, which regulates Gsp1p GTPase activity and thereby affects nucleocytoplasmic transport and cytoskeletal dynamics | 3 (2,1,2) | 1 (1) |  |
| Orf19.6975 | Yst1 | Ribosome-associated protein; antigenic in mice | 4 (3,4,5,3) | 2 (2,1) |  |
| Orf19.3618 | Ywp1 | Secreted yeast wall protein | 3 (2,1,1) | 3 (1,1,1) | Yes |
| Orf19.2709 | Zuo1 | Protein similar to *S. cerevisiae* Zuo1p, which is a cytosolic ribosome-associated chaperone | 3 (4,3,4) | 1 (1) |  |
| Orf19.4754 | Zwf1 | Putative glucose-6-phosphate dehydrogenase; antigenic in murine infection | 3 (1,1,2) | 2 (3,1) |  |

a) Identifier, protein name, description and signal peptide from Candida Genome Database (CGD) (Inglis DO. *et al.* Nucleic Acids Res. 2012).

Proteins indicated with * are considered as moonlighting (Nombela C. *et al.* Trends Microbiol. 2006; Chaffin WL. Microbiol Mol Biol Rev. 2008).

b) Proteins were included if they were identified in at least two replicates of one condition with at least 2 peptides in one replicate. There are four replicates of Normal Serum (NS) and three replicates of Heat Inactivated Serum (HIS). Dark grey background for proteins identified in all biological replicates and light grey background for proteins identified in 6 out of the 7 biological replicates. Noteworthy, the ~33% of C. albicans proteins were identified in at least 6 biological replicates.
