# Supplementary material for: Candida albicans Shaving to Profile Human Serum Proteins on Hyphal Surface
Source: Front Microbiol. 2015 Dec 8;6:1343. doi: 10.3389/fmicb.2015.01343 (PMC4672057; doi:10.3389/fmicb.2015.01343)
Supplement: Supplementary file 5 [file Table2.DOC]

**Table S2.** Human proteins not involved in complement and coagulation pathways identified on *C. albicans* surface after incubation with10% human serum.

| Accession number^a)^ | Gene symbol^a)^ | Description/Function^a)^ | | No. replicates  (peptides on each replicate)^b)^ | | |
| --- | --- | --- | --- | --- | --- | --- |
| **Metabolism** | | | | **NS** | | **HIS** |
| P63104 | YWHAZ | 14-3-3 protein zeta/delta | | 0 | | 2 (5,2) |
| P62917 | RPL8 | 60S ribosomal protein L8 | | 3 (1,1,1) | | 2 (1,2) |
| P11021 | GRP78 | 78 kDa glucose-regulated protein | | 3 (3,3,4) | | 2 (3,4) |
| P61204 | ARF3 | ADP-ribosylation factor 3 | | 4 (1,2,3,2) | | 0 |
| P02647 | ApoAI | Apolipoprotein A-I | | 4 (17,14,18,29) | | 3 (23,27,53) |
| P02652 | ApoAII | Apolipoprotein A-II | | 4 (4,4,7,8) | | 3 (6,7,9) |
| P06727 | ApoAIV | Apolipoprotein A-IV | | 4 (17,13,7,12) | | 3 (15,34,43) |
| Q6Q788 | ApoAV | Apolipoprotein A-V | | 3 (3,2,3) | | 2 (1,2) |
| P04114 | ApoB100 | Apolipoprotein B-100 | | 4 (102,97,240,296) | | 3 (281,209,322) |
| P02654 | ApoCI | Apolipoprotein C-I | | 4 (2,2,1,2) | | 3 (2,2,11) |
| P02655 | ApoCII | Apolipoprotein C-II | | 3 (1,4,1) | | 1 (6) |
| P02656 | ApoCIII | Apolipoprotein C-III | | 4 (1,1,3,2) | | 3 (1,2,7) |
| P55056 | ApoCIV | Apolipoprotein C-IV | | 4 (1,1,3,4) | | 2 (1,3) |
| P02649 | ApoE | Apolipoprotein E | | 4 (13,13,17,20) | | 3 (21,17,35) |
| Q13790 | ApoF | Apolipoprotein F | | 2 (2,3) | | 3 (2,2,5) |
| O14791 | ApoL1 | Apolipoprotein L1 | | 4 (1,2,16,16) | | 3 (15,11,28) |
| O95445 | ApoM | Apolipoprotein M | | 3 (1,2,4) | | 3 (5,5,7) |
| P08519 | Apo(a) | Apolipoprotein (a) | | 2 (4,11) | | 2 (21,5) |
| Q96KN2 | CNDP1 | Beta-Ala-His dipeptidase | | 0 | | 2 (2,12) |
| P43251 | BTD | Biotinidase | | 0 | | 3 (2,2,3) |
| P15169 | CPN1 | Carboxypeptidase N catalytic chain | | 2 (4,5) | | 3 (11,9,10) |
| P08311 | CTSG | Cathepsin G | | 2 (1,2) | | 0 |
| P00450 | CP | Ceruloplasmin; Ferroxidase | | 4 (16,13,3,10) | | 3 (14,26,38) |
| P11597 | CETP | Cholesteryl ester transfer protein | | 4 (8,5,15,11) | | 3 (14,14,14) |
| P68104 | EF1A | Elongation factor 1-alpha 1 | | 4 (5,3,1,3) | | 2 (3,4) |
| P13639 | EF2 | Elongation factor 2 | | 2 (2,1) | | 1 (1) |
| P22352 | GPX3 | Glutathione peroxidase 3 | | 4 (4,4,2,6) | | 3 (3,4,6) |
| P04406 | GAPDH | Glyceraldehyde-3-phosphate dehydrogenase | | 4 (2,4,8,4) | | 3 (2,4,2) |
| P11142 | HSPA8 | Heat shock cognate 71 kDa protein | | 3 (4,5,4) | | 1 (5) |
| P69905 | HBA2 | Hemoglobin subunit alpha | | 4 (2,3,3,4) | | 3 (5,7,9) |
| P35858 | ALS | Insulin-like growth factor-binding protein complex acid labile subunit | | 4 (3,2,8,17) | | 3 (18,14,21) |
| P51884 | LUM | Keratan sulfate proteoglycan lumican | | 0 | | 3 (6,1,5) |
| P30041 | PRDX6 | Peroxiredoxin-6 | | 2 (1,3) | | 1 (1) |
| P04180 | LCAT | Phosphatidylcholine-sterol acyltransferase | | 2 (9,8) | | 3 (4,4,5) |
| P55058 | PLPT | Phospholipid transfer protein | | 4 (7,7,8,11) | | 3 (7,6,8) |
| Q92954 | PRG4 | Proteoglycan 4 | | 4 (2,3,9,14) | | 2 (3,7) |
| P02787 | TF | Serotransferrin; Beta-1 metal-binding globulin | | 3 (29,26,2) | | 3 (19,11,28) |
| P02768 | ALB | Serum albumin | | 4 (52,54,27,30) | | 3 (44,46,43) |
| P35542 | SAA4 | Serum amyloid A-4 protein; Major acute phase reactant; Part of the HDL complex | | 4 (1,1,5,5) | | 2 (2,7) |
| P37837 | TAL | Transaldolase | | 4 (1,1,1,2) | | 2 (3,1) |
| P02774 | GC | Vitamin D-binding protein | | 3 (6,7,1) | | 3 (9,6,16) |
| **Immunoglobulins** | | | | **NS** | | **HIS** |
| P01876 | IGHA1 | Ig alpha-1 chain C region | | 4 (16,16,12,12) | | 3 (14,12,8) |
| P01877 | IGHA2 | Ig alpha-2 chain C region | | 3 (12,11,9) | | 1 (10) |
| P01880 | IGHD | Ig delta chain C region | | 4 (2,2,4,4) | | 2 (3,9) |
| P01857 | IGHG1 | Ig gamma-1 chain C region | | 4 (13,12,5,7) | | 3 (9,11,8) |
| P01859 | IGHG2 | Ig gamma-2 chain C region | | 4 (11,8,2,5) | | 3 (5,5,7) |
| P01860 | IGHG3 | Ig gamma-3 chain C region (HDC); Heavy chain disease protein | | 4 (10,9,4,11) | | 3 (11,8,6) |
| P01861 | IGHG4 | Ig gamma-4 chain C region | | 4 (8,7,2,5) | | 1 (8) |
| P01766 | - | Ig heavy chain V-III region BRO | | 3 (3,2,2) | | 3 (3,2,4) |
| P01767 | - | Ig heavy chain V-III region BUT | | 2 (3,2) | | 1 (3) |
| P01768 | - | Ig heavy chain V-III region CAM | | 3 (2,1,2) | | 1 (3) |
| P01769 | - | Ig heavy chain V-III region GA | | 2 (2,1) | | 0 |
| P01781 | - | Ig heavy chain V-III region GAL | | 2 (3,2) | | 3 (3,3,5) |
| P01765 | - | Ig heavy chain V-III region TIL | | 3 (3,1,2) | | 2 (3,3) |
| P01764 | - | Ig heavy chain V-III region VH26 | | 1 (2) | | 2 (3,4) |
| P01834 | IGKC | Ig kappa chain C region | | 4 (9,8,6,7) | | 3 (7,6,2) |
| P01617 | - | Ig kappa chain V-II region TEW | | 2 (2,2) | | 0 |
| P01619 | - | Ig kappa chain V-III region B6 | | 4 (1,1,1,1) | | 2 (2,1) |
| P01623 | - | Ig kappa chain V-III region WOL | | 2 (3,6) | | 1 (6) |
| P01842 | IGLC1 | Ig lambda chain C regions | | 3 (7,4,6) | | 0 |
| P04208 | - | Ig lambda chain V-I region WAH | | 2 (2,2) | | 1 (1) |
| P0CG05 | IGLC2 | Ig lambda-2 chain C regions | | 1 (4) | | 3 (8,6,4) |
| P01871 | IGHM | Ig mu chain C region | | 4 (22,21,11,17) | | 3 (16,19,12) |
| P04220 | - | Ig mu heavy chain disease protein | | 3 (13,13,9) | | 2 (12,12) |
| P01591 | IGJ | Immunoglobulin J chain; Serves to link two monomer units of either IgM or IgA | | 4 (4,5,1,5) | | 2 (3,2) |
| B9A064 | IGLL5 | Immunoglobulin lambda-like polypeptide 5 | | 0 | | 2 (5,5) |
| **Cytoskeleton** | | | | **NS** | | **HIS** |
| P68032 | ACTC1 | Actin, alpha cardiac muscle 1 | | 1 (4) | | 3 (10,9,10) |
| P60709 | ACTB | Actin, cytoplasmic 1 | | 4 (6,5,3,4) | | 2 (15,15) |
| P12814 | ACTN1 | Alpha-actinin-1; F-actin cross-linking protein | | 0 | | 2 (1,5) |
| P16157 | ANK1 | Ankyrin-1 | | 2 (16,20) | | 0 |
| P02730 | AE1 | Band 3 anion transport protein | | 2 (11,11) | | 1 (1) |
| Q562R1 | ACTBL2 | Beta-actin-like protein 2 | | 2 (2,3) | | 2 (5,5) |
| P33151 | CDH5 | Cadherin-5 | | 2 (3,5) | | 2 (5,1) |
| P16452 | EPB42 | Erythrocyte membrane protein band 4.2 | | 2 (2,3) | | 0 |
| P27105 | EPB72 | Erythrocyte band 7 integral membrane protein | | 2 (2,1) | | 1 (1) |
| P21333 | FLN | Filamin-A; Actin binding protein 280 | | 2 (1,1) | | 3 (8,10,20) |
| P06396 | GSN | Gelsolin; Actin-depolyperizing factor | | 4 (9,10,42,41) | | 3 (35,27,34) |
| P08514 | GP2B | Integrin alpha-IIb; Platelet membrane glycoprotein IIb | | 0 | | 3 (5,4,4) |
| P05106 | GP3A | Integrin beta-3; Platelet membrane glycoprotein IIIa | | 1 (1) | | 2 (2,3) |
| P13645 | KRT10 | Keratin, type I cytoskeletal 10 | | 3 (18,8,2) | | 3 (3,7,2) |
| P35527 | KRT9 | Keratin, type I cytoskeletal 9 | | 3 (12,8,9) | | 3 (10,12,2) |
| P04264 | KRT1 | Keratin, type II cytoskeletal 1 | | 4 (22,15,4,12) | | 3 (10,14,7) |
| P35908 | KRT2 | Keratin, type II cytoskeletal 2 epidermal | | 4 (10,5,3,4) | | 3 (2,7,7) |
| P04259 | KRT6B | Keratin, type II cytoskeletal 6B | | 2 (3,3) | | 0 |
| P08567 | PLEK | Pleckstrin; Platelet 47 kDa protein | | 0 | | 2 (1,3) |
| P07737 | PFN1 | Profilin-1 | | 0 | | 3 (2,1,4) |
| P02549 | SPTA | Spectrin alpha chain, erythrocyte | | 2 (29,27) | | 0 |
| P11277 | SPTB | Spectrin beta chain, erythrocyte | | 2 (21,23) | | 0 |
| Q9Y490 | TLN1 | Talin-1 | | 2 (3,17) | | 3 (17,22,16) |
| P68366 | TUBA4A | Tubulin alpha-4A chain | | 0 | | 2 (2,3) |
| P07437 | TUBB | Tubulin beta chain | | 3 (2,1,2) | | 1 (4) |
| P18206 | VCL | Vinculin | | 0 | | 3 (1,3,10) |
| **Others** | | | | **NS** | | **HIS** |
| P27348 | YWHAQ | | 14-3-3 protein theta | | 3 (2,1,1) | 0 |
| Q9HDC9 | APMAP | | Adipocyte plasma membrane-associated protein | | 2 (1,4) | 1 (2) |
| P43652 | AFM | | Afamin; Vitamin E binding protein | | 0 | 3 (5,11,17) |
| P01011 | SERPINA3 | | Alpha-1-antichymotrypsin | | 2 (1,2) | 3 (27,19,30) |
| P04217 | A1BG | | Alpha-1B-glycoprotein | | 2 (1,1) | 3 (16,15,16) |
| P02765 | AHSG | | Alpha-2-HS-glycoprotein; Promotes endocytosis, possesses opsonic properties | | 4 (6,5,10,11) | 3 (10,10,5) |
| Q8NI99 | AGF | | Angiopoietin-related protein 6 | | 2 (2,3) | 0 |
| P01019 | AGT | | Angiotensinogen (Serpin A8) | | 4 (6,5,5,5) | 3 (11,13,19) |
| P58335 | ANTXR2 | | Anthrax toxin receptor 2; Necessary for cellular interactions with laminin and the extracellular matrix | | 2 (1,3) | 2 (3,4) |
| P22792 | CPN2 | | Carboxypeptidase N subunit 2 | | 3 (1,9,11) | 3 (11,7,9) |
| O43866 | CD5L | | CD5 antigen-like | | 4 (10,10,3,8) | 3 (9,4,2) |
| Q9Y6Z7 | COLEC10 | | Collectin-10; Lectin that binds to various sugars | | 3 (3,3,1) | 0 |
| P08185 | SERPINA6 | | Corticosteroid-binding globulin | | 0 | 3 (11,8,12) |
| Q12805 | EFEMP1 | | EGF-containing fibulin-like extracellular matrix protein 1 | | 3 (1,8,12) | 3 (6,1,3) |
| Q16610 | ECM1 | | Extracellular matrix protein 1 | | 2 (2,2) | 0 |
| Q86UX7 | FERMT3 | | Fermitin family homolog 3; Plays a central role in cell adhesion in hematopoietic cells | | 3 (1,1,5) | 2 (1,3) |
| Q9UGM5 | FETUB | | Fetuin-B; Protease inhibitor required for egg fertilization | | 3 (1,8,8) | 3 (7,3,5) |
| P23142 | FBLN1 | | Fibulin-1; Incorporated into fibronectin-containing matrix fibers | | 4 (1,3,9,20) | 3 (12,11,3) |
| Q08380 | M2BP | | Galectin-3-binding protein; Modulate cell-cell and cell-matrix interactions | | 3 (10,12,1) | 0 |
| P00738 | HP | | Haptoglobin; Captures free plasma hemoglobin; Antimicrobial; Antioxidant | | 2 (19,16) | 3 (20,19,24) |
| P00739 | HPR | | Haptoglobin-related protein | | 4 (17,14,11,12) | 3 (15,17,22) |
| P34931 | HSPA1L | | Heat shock 70 kDa protein 1-like | | 3 (4,5,3) | 2 (3,3) |
| P54652 | HSPA2 | | Heat shock-related 70 kDa protein 2 | | 1 (3) | 2 (4,4) |
| P17066 | HSPA6 | | Heat shock 70 kDa protein 6 | | 2 (4,4) | 1 (5) |
| P08238 | HSP90B | | Heat shock protein HSP 90-beta | | 3 (2,2,2) | 0 |
| P02790 | HPX | | Hemopexin; Binds heme group | | 3 (5,7,1) | 3 (6,6,21) |
| P26927 | MST1 | | Hepatocyte growth factor-like protein | | 0 | 2 (10,4) |
| Q96KK5 | HIST1H2AH | | Histone H2A type 1-H | | 2 (3,2) | 0 |
| O60814 | HIST1H2BK | | Histone H2B type 1-K | | 2 (3,2) | 1 (1) |
| P62805 | HIST1H4 | | Histone H4 | | 2 (6,8) | 0 |
| Q86YZ3 | HRNR | | Hornerin | | 0 | 2 (1,2) |
| P19827 | ITIH1 | | Inter-alpha-trypsin inhibitor heavy chain H1 | | 4 (5,8,23,20) | 3 (31,31,35) |
| P19823 | ITIH2 | | Inter-alpha-trypsin inhibitor heavy chain H2 | | 4 (3,6,22,25) | 3 (41,35,44) |
| Q06033 | ITIH3 | | Inter-alpha-trypsin inhibitor heavy chain H3 | | 2 (4,11) | 3 (15,14,18) |
| Q14624 | ITIH4 | | Inter-alpha-trypsin inhibitor heavy chain H4 | | 4 (23,22,37,42) | 3 (50,45,43) |
| P29622 | SERPINA4 | | Kallistatin | | 2 (5,10) | 3 (13,19,16) |
| P02750 | LRG1 | | Leucine-rich alpha-2-glycoprotein | | 0 | 3 (10,6,9) |
| P18428 | LBP | | Lipopolysaccharide-binding protein | | 4 (8,9,13,14) | 3 (17,16,15) |
| Q8TDL5 | BPIFB1 | | Long palate, lung and nasal epithelium carcinoma-associated protein 1 | | 3 (1,3,6) | 2 (4,2) |
| P08571 | CD14 | | Monocyte differentiation antigen CD14; In concert with LBP, binds to monomeric LPS | | 2 (7,10) | 3 (6,5,3) |
| Q96PD5 | PGRPL | | N-acetylmuramoyl-L-alanine amidase | | 3 (1,13,16) | 3 (13,5,11) |
| P02776 | PF4 | | Platelet factor 4; Released during platelet aggregation | | 4 (2,2,1,3) | 2 (3,1) |
| P36955 | SERPINF1 | | Pigment epithelium-derived factor | | 1 (1) | 3 (11,10,19) |
| Q9UHG3 | PCL1 | | Prenylcysteine oxidase 1 | | 0 | 3 (5,7,3) |
| P02760 | AMBP | | Inter-alpha-trypsin inhibitor; inhibits trypsin, plasmin and lysosomal granulocytic elastase | | 4 (9,7,5,11) | 3 (17,17,4) |
| P21980 | TGM2 | | Protein-glutamine gamma glutamyl-transferease 2 | | 2 (1,2) | 0 |
| A6NIZ1 | - | | Ras-related protein Rap 1b like protein | | 2 (1,2) | 1 (1) |
| P02753 | RBP4 | | Plasma retinol-binding protein 4; RBP binds retinol and interact with transthyretin | | 0 | 2 (3,5) |
| Q13103 | SPP2 | | Secreted phosphoprotein 24 | | 3 (1,1,1) | 3 (2,13) |
| P49908 | SEPP1 | | Selenoprotein P | | 3 (3,2,1) | 1 (3) |
| P27169 | PON1 | | Serum paraoxonase/arylesterase 1 | | 4 (1,3,12,15) | 3 (16,14,17) |
| Q15166 | PON3 | | Serum paraoxonase/lactonase 3 | | 1 (2) | 3 (3,3,6) |
| P04278 | SHBG | | Sex hormone-binding globulin | | 4 (5,3,17,11) | 3 (12,10,9) |
| O00391 | QSOX1 | | Sulfhydryl oxidase 1 | | 2 (7,4) | 1 (3) |
| P07996 | THBS1 | | Thrombospondin-1; Binds heparin | | 4 (2,3,24,46) | 3 (31,25,19) |
| P05543 | SERPINA7 | | Thyroxine binding globulin (TBG) | | 2 (2,1) | 3 (6,4,14) |
| P02766 | TTR | | Transthyretin | | 4 (3,3,6,8) | 3 (8,5,12) |
| Q8WUA8 | TSKU | | Tsukushin | | 4 (1,1,2,1) | 0 |

a) Function from UniProtKB/Swiss-Prot database (UniProt, Consortium. [Nucleic Acids Res.](http://dx.doi.org/doi:10.1093/nar/gkt1140) 2014).

b) Proteins were included if they were identified in at least two replicates with at least 2 peptides in one replicate of one condition. There are four replicates of Normal Serum (NS) and three replicates of Heat Inactivated Serum (HIS).
