# Supplementary material for: Candida albicans Shaving to Profile Human Serum Proteins on Hyphal Surface
Source: Front Microbiol. 2015 Dec 8;6:1343. doi: 10.3389/fmicb.2015.01343 (PMC4672057; doi:10.3389/fmicb.2015.01343)
Supplement: Supplementary file 6 [file Table3.DOC]

**Table S3. Comparison of proteins identified on *C. albicans* cell surface incubated with 10% human serum with yeasts and hyphae grown in Lee´s medium by shaving approach.**

| **In all conditions**  **(yeasts, hyphae with and without human serum)^a)^** | **Hyphal specific**  **(hyphae with and without human serum)^a)^** | **Only in hyphae with human serum^a)^** |
| --- | --- | --- |
| 304 proteins | 55 proteins | 12 proteins |
| Abp1, Acb1, Acc1, Acs2, Act1, Ade12, Ade17, Adh1, Adh2, Adk1, Ado1, Agm1, Ahp1, Ala1, Als1, Anb1, Ape2, Arg1, Aro4, Aro8, Asc1, Asn1, Asr1, Asr3, Atp1, Atp2, Bat22, Bcy1, Bfr1, Bgl2, Bmh1, Cam1, Cam1-1, Car2, Cdc12, Cdc19, Cdc3, Cdc42, Cdc48, Cdc60, Cef3, Cht2, Cht3, Cmd1, Cof1, Coi1, Crh11, Csp37, Cyp1, Dak2, Ddr48, Ded1, Ded81, Dps1-1, Ecm33, Efb1, Eft2, Egd1, Egd2, Eif4e, Emp46, Eng1, Eno1, Erg10, Erg13, Faa4, Fas1, Fas2, Fba1, Fdh3, Gda1, Gfa1, Gis2, Glc7, Gln1, Glt1, Glx3, Gnd1, Gpd2, Gph1, Gre3, Grp2, Grs1, Gsp1, Hem13, Hgt6, Hmo1, Hnt1, Hom6, Hsp104, Hsp12, Hsp21, Hsp70, Hsp90, Hxk2, Idi1, Ihd1, Ino1, Ipp1, Ist2, Kar2, Krs1, Lat1, Lsc1, Lsp1, Lys22, Mbf1, Mdg1, Mdh1, Mdh1-1, Met15, Met6, Mis11, Mlc1, Mnt1, Mp65, Msb2, Msi3, Mvd, Nhp6a, Nop1, Orf19.1052, Orf19.1086, Orf19.1564, Orf19.1862, Orf19.1946, Orf19.200, Orf19.2125, Orf19.2269, Orf19.2286, Orf19.2296, Orf19.2478.1, Orf19.2769, Orf19.3037, Orf19.3354, Orf19.3475, Orf19.3572.3, Orf19.3690.2, Orf19.3932, Orf19.4149.1, Orf19.4216, Orf19.4246, Orf19.5281, Orf19.5620, Orf19.5773, Orf19.5943.1, Orf19.6160, Orf19.6403, Orf19.6415.1, Orf19.6507, Orf19.6553, Orf19.6701, Orf19.6882.1, Orf19.7085, Orf19.7297, Pck1, Pda1, Pdc11, Pdi1, Pet9, Pfk1, Pfk2, Pfy1, Pga4, Pgi1, Pgk1, Pgm2, Phr1, Phr2, Pil1, Pir1, Plb4.5, Pma1,Pmi1, Pmm1, Prx1, Ras1, Rbp1, Rbt1, Rbt5, Rct1, Rdi1, Rhd3, Rho1, Rhr2, Rnr21, Rpl10, Rpl10A, Rpl11, Rpl12, Rpl13, Rpl14, Rpl15A, Rpl17B, Rpl18, Rpl19A, Rpl2, Rpl20B, Rpl21A, Rpl23A, Rpl24A, Rpl25, Rpl27A, Rpl28, Rpl3, Rpl30, Rpl32, Rpl35, Rpl38, Rpl39, Rpl4B, Rpl5, Rpl6, Rpl8B, Rpl9B, Rpp0, Rps1, Rps10, Rps12, Rps13, Rps14B, Rps15, Rps16A, Rps17B, Rps18, Rps19A, Rps20, Rps21, Rps21B, Rps23A, Rps24, Rps25B, Rps26A, Rps27, Rps28B, Rps3, Rps5, Rps6A, Rps7A, Rps8A, Rps9B, Sah1, Sam2, Sap9, Sar1, Sbp1, Ser33, Ses1, Sgt1, Sgt2, Sik1, Sim1, Skp1, Slk19, Smt3, Snz1, Sod1, Srb1, Ssa2, Ssb1, Ssc1, Ssr1, Ssz1, Sti1, Sub2, Sui2, Sur7, Tal1, Tdh3, Thr4, Tif, Tif3, Tif4631, Tkl1, Tma19, Tom70, Tos1, Tpi1, Tpm2, Trr1, Trx1, Tsa1, Ttr1, Tub1, Tub2, Tup1, Uba1, Ubi3, Ugp1, Ura2, Utr2, Vas1, Vma4, Wh11, Xyl2, Ybn5, Ynk1, Yps7, Ypt1, Ypt31, Yrb1, Yst1, Ywp1, Zuo1 | Als3, Arf2, Aro1, Cct7, Chc1, Cip1, Cys3, Ece1, Frs1, Frs2, Gad1, Gca1, Gsc1, Het1, Hhf1, Hta1, Hta2, Hyr1, Idp1, Idp2, Kex2, Kin2, Mnn26, Mxr1, Myo2, Orf19.1376, Orf19.3003, Orf19.3681, Orf19.4796, Orf19.5682, Orf19.7368, Orf19.86, Pga10, Pga45, Pga63, Plb3, Pmt2, Pr26, Pst1, Pst3, Ret2, Rpl82, Rpp2A, Rvs161, Sec2, Sec24, Sec26, Sec4, Ser1, Shm2, Sod5, Vps1, Ydj1, Ykt6, Zwf1 | Bzz1, Glk4, Hht21, Hta3, Orf19.3053, Orf19.3061.1, Orf19.3247, Orf19.3499, Orf19.8, Pra1, Sap5, Tef1 |

a) Protein names are from *Candida* Genome Database (CGD) (Inglis DO. *et al.* Nucleic Acids Res. 2012).

Yeast and hyphal forms were grown in Lee media at pH 4.2 and pH 6.7, respectively (Gil-Bona A. *et al.* J. Proteomics 2015). Trypsin treatment was done with 5 μg and 5 min of incubation at 37ºC. Nup is the only protein common between yeasts and hyphae induced with human serum, not identified in hyphae without human serum.

Mass spectrometry proteomics data (.raw files) has been deposited in *Candida albicans* PeptideAtlas with the data set identifier PASS00446 for yeasts and hyphae growth in Lee´s medium with and without 10% human serum (Vialas V. *et al.* J. Proteomics 2014).
